# The TRIM3/TLR3 axis overrides IFN-β feedback inhibition to suppress NSCLC progression

**DOI:** 10.1038/s41419-025-08265-w

**Published:** 2026-01-16

**Authors:** Jianyu Xu, Qianfang Hu, Ying Zhu, Qian Liu, Feng Wang, Yanxia Yu, Wenjuan Wang, Xinyuan Ding

**Affiliations:** 1 Department of Pharmacy, Medical Science and Technology Innovation Center, The Affiliated Suzhou Hospital of Nanjing Medical University, Suzhou Municipal Hospital, Gusu School of Nanjing Medical University, Suzhou, China; 2 Department of Pathology, The Affiliated Suzhou Hospital of Nanjing Medical University, Suzhou, China; 3 Department of Pharmacy, Children’s Hospital of Soochow University, Suzhou, China

**Keywords:** Non-small-cell lung cancer, Ubiquitylation

## Abstract

Interferon-beta (IFN-β) has potent antitumor activity, but its clinical therapeutic potential is undermined by intrinsic negative feedback loops that suppress IFN-β production. However, the feedback mechanisms regulating IFN-β homeostasis in non-small cell lung cancer (NSCLC) remain unclear. We found that tripartite motif containing 3 (TRIM3) promotes the transcription and mRNA expression of *IFNB1*. Conversely, excessive IFN-β inhibits expression of TRIM3, creating their reciprocal feedback loop. Mass spectrometry revealed that toll-like receptor 3 (TLR3), a key sensor that triggers IFN-β production, is the interacting partner of TRIM3. Following the elucidation of the interactive mode between TRIM3 and TLR3, we found that activation of the TRIM3/TLR3 axis induced IFN-β secretion and overrode the feedback inhibition. Sustained IFN-β secretion subsequently inhibits NSCLC cell proliferation and reprograms the tumor microenvironment by increasing the infiltration levels of CD4^+^ T cells, M1 macrophages and NK cells. Our findings revealed a reciprocal negative feedback loop in the regulation of IFN-β signaling, highlighting the role of the TRIM3/TLR3 axis in the suppression of NSCLC progression and offering a promising strategy to suppress tumor growth and enhance immunotherapy efficacy in NSCLC.

## Introduction

Non-small cell lung cancer (NSCLC) remains a leading cause of cancer-related mortality worldwide, accounting for 85% of all lung malignancies. Despite advances in targeted therapies and immunotherapies, the 5-year survival rate for advanced NSCLC remains below 20%, underscoring the urgent need to elucidate novel molecular targets involved in tumor progression [1–3]. Emerging evidence suggests that dysregulation of tumor immune microenvironment signaling, particularly involving cytokine networks, plays a pivotal role in NSCLC pathogenesis and therapeutic resistance [4–6].

Interferons (IFNs) are potent cytokines with tumor-suppressive properties that function as critical coordinators of anticancer immunity [7, 8]. As a subclass of type I IFNs, IFN-β directly inhibits tumor cell proliferation and survival while mobilizing systemic immune responses through enhanced antigen presentation, natural killer (NK) cell activation, and T-cell effector function [9–11]. However, its clinical efficacy in oncology remains suboptimal. In a Phase III clinical trial in patients with NSCLC, compared with a control treatment, IFN-β therapy did not yield a statistically significant survival advantage [12]. This result could arise from the negative feedback inhibition, which modulates IFN-β signaling to prevent hyperactivation [13, 14]. IFN-β secretion is governed primarily by toll-like receptors (TLRs) [15]. However, the feedback and molecular mechanisms governing IFN-β signaling in NSCLC remain unclear.

The ubiquitin-proteasome system is a key posttranslational modification pathway in cellular processes. E3 ubiquitin ligases and deubiquitinating enzymes alter protein localization and stability within cells [16]. Dysregulation of this system is closely linked to the development and progression of cancer. Many studies have indicated an interplay between ubiquitin-modifying enzymes and interferons, where proteins such as ubiquitin-specific peptidase 18 (USP18), an interferon-inducible protein, offer negative feedback on type I interferon signaling [17–19].

Tripartite motif containing 3 (TRIM3), a highly conserved E3 ubiquitin ligase within the TRIM protein family, has garnered increasing attention in cancer research. Studies have demonstrated that TRIM3 suppresses glioblastoma by inhibiting c-Myc transcription, impedes hepatocellular carcinoma metastasis, and curbs cervical cancer progression [20, 21]. However, its molecular mechanisms in NSCLC remain poorly defined, particularly regarding interactions with immune modulation.

In this study, we identified the TRIM3/TLR3 axis as a critical regulator of IFN-β signaling in NSCLC. Although TRIM3 promotes the expression of *IFNB1*, excessive IFN-β may ultimately suppress TRIM3 expression, leading to feedback inhibition. Notably, activation of the TRIM3/TLR3 axis induced sustained IFN-β secretion, overriding the feedback inhibition. Increased IFN-β secretion inhibits NSCLC cell proliferation and increases the infiltration levels of CD4^+^ T cells, M1 macrophages and NK cells into the tumor microenvironment, consequently inhibiting NSCLC progression. Our study reveals a novel therapeutic theory for IFN-β-based antitumor therapy, suggesting that disrupting this reciprocal negative feedback loop enables new NSCLC treatment strategies.

## Materials and methods

### Cell culture and treatment

Human NSCLC cell lines (A549, NCI-H1299, PC-9, NCI-H2030, NCI-H2228, 95-D, and NCI-H1944), BEAS-2B cells, HEK293T cells, and murine lewis lung carcinoma (LLC) cells were obtained from the Cell Bank of the Chinese Academy of Sciences. All the cell lines were authenticated by short tandem repeat (STR) profiling and tested for mycoplasma contamination. Cells were cultured in RPMI-1640 or DMEM medium supplemented with 10% fetal bovine serum and maintained at 37 °C in a humidified atmosphere containing 5% CO₂. CRISPR-Cas9 knockout and overexpression were performed using custom-designed constructs. For knockout, sgRNA sequences (Tables S1, 2) were cloned and inserted into the lentiguide backbone vector. For overexpression, target genes were inserted into the pLV3 expression vector. Viruses were packaged using the 2nd-generation vector system, with HEK293T cells transfected with psPAX2, pMD2.G retroviral packaging plasmids. Lentivirus-infected cells were selected using hygromycin for knockout and puromycin for overexpression to establish stable genetic models. The knockout and overexpression efficiency were verified by western blotting. Cultured cells were treated with IFN-β (TMPY-03145, TargetMol, Massachusetts, USA), poly(I:C) (T12516, TargetMol) and BMS-986165 (T14687, TargetMol).

### RNA extraction and quantitative real-time PCR (qPCR)

Total RNA was isolated from cultured cells using TRIzol reagent (15596026CN, Invitrogen, California, USA) following the manufacturer’s protocol. For cDNA synthesis, 1 μg of total RNA was reverse transcribed using a PrimeScript RT Reagent Kit (R212-01, Vazyme, Nanjing, China). qPCR was performed using a qPCR Master Mix Kit (Q712-02, Vazyme) with species-specific primers. The primer sequences are listed in Table S3.

### Western blotting (WB)

Western blotting was performed in A549 and NCI-H1299 cell lines as previously described [22]. The primary antibodies used for incubation included those against GAPDH (#2118, Cell Signaling Technology, Massachusetts, USA), TRIM3 (PU791217, Abmart, Shanghai, China), DDDDK tag (#14793, Cell Signaling Technology), Myc tag (#2276, Cell Signaling Technology), HA tag (#3724, Cell Signaling Technology), IRF3 (T55779, Abmart), phospho-IRF3 (TA2436, Abmart), TBK1 (TD7026, Abmart), phospho-TBK1 (T58364, Abmart), TLR3 (ab307442, Abcam) and IFN-β (PA6218, Abmart).

### Colony formation and cell counting kit-8 (CCK-8) assays

Cellular proliferation was assessed using a cell counting kit-8 (CK04, Dojindo, Kumamoto, Japan) according to the manufacturer’s protocol. For the colony formation assays, A549, NCI-H1299 and LLC cells were trypsinized, resuspended in complete medium, and seeded at a density of 1000 cells per well in 6-well plates. The cells were maintained under standard culture conditions (37 °C, 5% CO₂) for 7–10 days until visible colonies formed. Colonies were fixed with 4% paraformaldehyde and stained using a Wright-Giemsa Stain Kit purchased from Nanjing Jiancheng Bioengineering Institute (D010-1, Nanjing, China). Stained colonies (>50 cells per cluster) were quantified.

### Proximity ligation assay (PLA)

A549 and NCI-H1299 cells were seeded into confocal dishes and cultured under standard conditions. The cells were fixed with 4% paraformaldehyde, permeabilized with 0.1% Triton X-100, and subsequently blocked with 5% BSA. For primary antibody incubation, the cells were treated overnight at 4 °C with primary antibodies against TRIM3 (rabbit polyclonal, 1:200) and TLR3 (mouse monoclonal, 1:200). The following day, the samples were incubated at 37 °C for 60 min with the Mirus PLA probe (DUO92004, Sigma-Aldrich, State of Missouri, USA) and the Plus PLA probe (DUO92002, Sigma-Aldrich). Ligation was performed at 37 °C for 30 min, and amplification was performed for 100 min. The nuclei were labeled with DAPI.

### Immunohistochemistry (IHC)

Six primary NSCLC and adjacent normal tissue samples were obtained after acquiring informed consent from patients in the Affiliated Suzhou Hospital of Nanjing Medical University, excluding samples that are necrotic or have insufficient tumor content. The criteria were pre-established in previous study [22]. The samples were incubated with antibodies against CD4 (TB6525, Abmart), IFN-β (PA6218, Abmart), CD49b (PK22887, Abmart), CD86 (T55238, Abmart) and CD163 (#93498, Cell Signaling Technology) overnight at 4 °C. Sections were visualized using DAB chromogen. Two independent experts were blinded to assess each result.

### Syngeneic mouse model

A syngeneic mouse model was established by injecting 0.5 × 10⁶ LLC cells subcutaneously into the flanks of female 6-week-old C57BL/6 mice. Tumor-bearing mice received intraperitoneal poly(I:C) (1 mg/kg) injections every 3 days, with daily monitoring of body weight and tumor dimensions (volume = length × width² × 0.5). Mice were euthanized before the maximal tumor size reached 1500 mm^3^. After 15 days, mice were euthanized, tumors were excised, weighed and photographed. Sample size was chosen based on ethical considerations to minimize animal use, while ensuring feasibility. Mice were randomly assigned to each group, ensuring equal distribution based on body weight. All measurements were in a blinded manner.

### Bioinformatic analysis

Bioinformatic analysis was conducted using public databases. GSE226667 (GEO datasets, https://www.ncbi.nlm.nih.gov/) was analyzed for differential expression, with the top 500 genes intersecting 655 ubiquitin-proteasome system genes [23]. *TRIM3* expression in cytokine-stimulated mouse tumor models was retrieved from TISMO (http://tismo.cistrome.org/) [24], and mRNA expression, clinicopathological associations (UALCAN, https://ualcan.path.uab.edu/) [25], and survival curves (KM plotter, https://kmplot.com/) were evaluated [26]. Immune infiltration levels and pancancer expression patterns were explored via TIMER2.0 (http://timer.cistrome.org/) [27]. The prediction of ubiquitination-specific lysine residues was conducted via GPS-Uber (https://gpsuber.biocuckoo.cn/) [28].

### Statistical analysis

All data were obtained from at least four independently repeated experiments. Statistical analyses were performed using GraphPad Prism 9.0. Two-tailed Student’s *t* test were used for comparisons between two groups, and one-way ANOVA was used for comparisons among more than two groups. Numerical data are presented as the means ± standard error (SEM) to reflect data variability. Statistical significance was assigned to *p* values < 0.05.

## Results

### Identification of TRIM3 as a target for the interferon-stimulated response in NSCLC

**Fig. 1:**
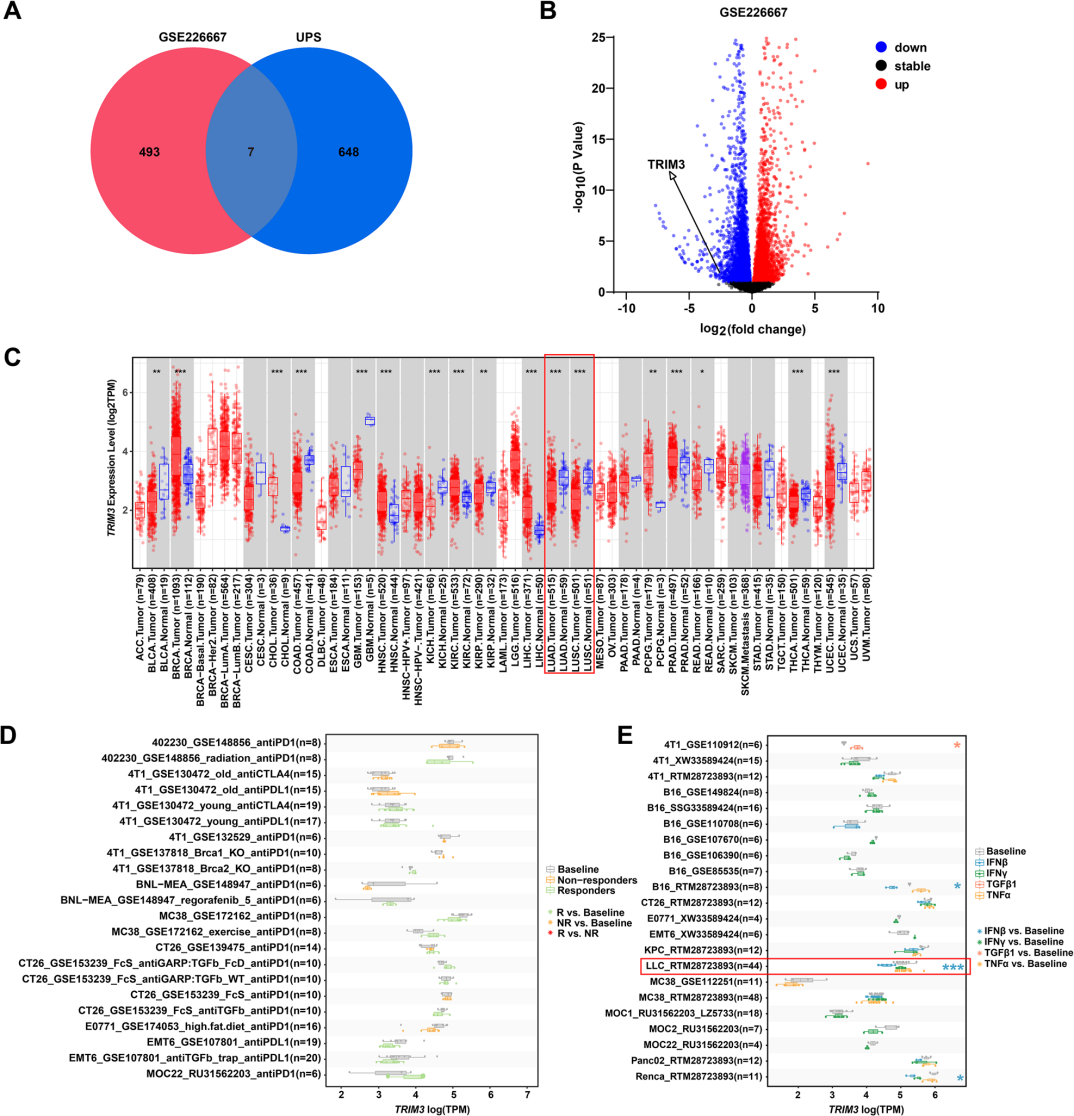
Identification of TRIM3 as a target for the interferon-stimulated response in NSCLC. **A** Venn diagram illustrating the intersection of the top 500 DEGs from GSE226667 with 655 known ubiquitin‒proteasome system (UPS) genes. **B** Volcano plot showing DEGs from the GSE226667 dataset. **C** Box plot analysis of *TRIM3* mRNA levels across cancers from the TCGA database. **D** Box plot showing changes of *TRIM3* mRNA expression in mouse tumor models treated with PD-1, PD-L1 and CTLA-4 inhibitors. **E** Box plot showing changes in *TRIM3* mRNA expression in mouse tumor models following cytokine treatment. *p < 0.05; **p < 0.01; ***p < 0.001.

To identify potential ubiquitin-modifying enzymes associated with interferon secretion, we screened RNA sequencing datasets from interferon-stimulated lung cancer cells in the GEO database. The GSE226667 dataset revealed genome-wide expression profiles of A549 lung cancer cells treated with composite IFNs [23]. We selected the top 500 differentially expressed genes (DEGs) and intersected them with 655 known ubiquitin-proteasome system-related genes, yielding seven interferon-responsive candidates (Fig. 1A, B). Among these genes, TRIM3 was prioritized for further study because of its most pronounced difference in expression in NSCLC (Fig. S1). The expression of *TRIM3* mRNA was also significantly downregulated in most cancer types, including lung adenocarcinoma (LUAD) and squamous cell carcinoma (LUSC) (Fig. 1C). In subcutaneous tumor models of diverse tumors, unlike the responses to PD-1, PD-L1 and CTLA-4 inhibitors or other cytokines, *TRIM3* mRNA expression decreased specifically upon IFN-β stimulation in the LLC lung cancer model (Fig. 1D, E). These findings suggest that TRIM3 exhibits a distinct response to IFN-β stimulation in NSCLC.

### Interaction between TRIM3 and IFN-β establishes a reciprocal feedback loop in NSCLC

**Fig. 2:**
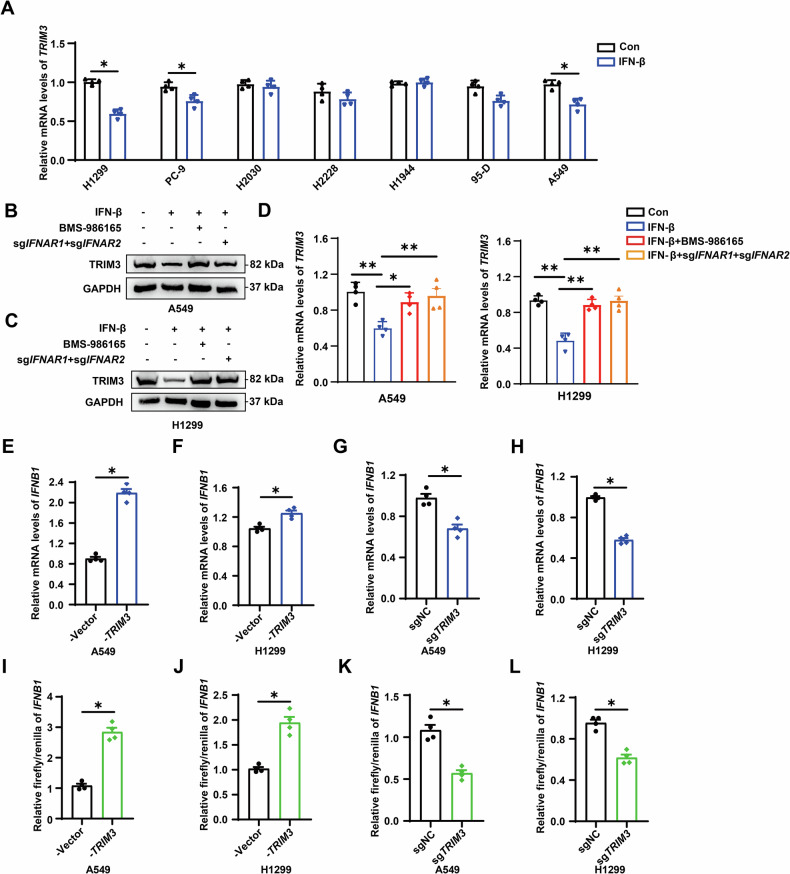
Interplay between TRIM3 and IFN-β establishes a reciprocal feedback loop in NSCLC. **A** *TRIM3* mRNA expression was analyzed by qPCR in H1299, PC-9, H2030, H2228, H1944, 95-D and A549 cells following stimulation with 100 ng/mL IFN-β (n = 4). Significance was assessed using Student’s *t* test. **B**–**D** TRIM3 protein and mRNA levels were measured in A549 and H1299 cells treated with IFN-β ± 20 ng/mL BMS-986165 or *IFNAR1* and *IFNAR2* knockout. Statistical significance was analyzed using one-way ANOVA. **E**–**H** *IFNB1* mRNA levels were quantified by qPCR in A549 and H1299 cells with TRIM3 modulation (n = 4). Significance was assessed using Student’s *t* test. **I**–**L** Luciferase reporter assays were performed in A549 and H1299 cells transfected with *IFNB1* promoter luciferase constructs (n = 4). Significance was assessed using Student’s *t* test. *p < 0.05; **p < 0.01.

Based on the finding that *TRIM3* mRNA expression was downregulated in response to IFN-β stimulation in LLC mouse models, we next performed in vitro experiments to validate the impact of IFN-β on TRIM3 expression. Following IFN-β stimulation, *TRIM3* mRNA expression was downregulated in H1299, PC-9, and A549 cells (Fig. 2A). The inhibition of the type I IFN pathway using the tyrosine kinase 2 (TYK2) inhibitor BMS-986165, as well as genetic knockout of interferon alpha and beta receptor subunit 1 (*IFNAR1*) and *IFNAR2*, reversed this suppression, restoring both TRIM3 protein and mRNA levels in A549, H1299 and PC-9 cells (Figs. 2B–D and S2A–C). Building on reported negative feedback between type I IFNs and ubiquitin-modifying enzymes [17–19], we hypothesized that TRIM3 might reciprocally regulate IFN-β production. Lentiviral knockout or overexpression of TRIM3 (Fig. S2D–F) demonstrated that increased TRIM3 expression increased *IFNB1* mRNA levels, whereas its depletion reduced *IFNB1* levels in A549, H1299 and PC-9 cells (Figs. 2E–H and S2G). Luciferase reporter assays further confirmed the TRIM3-dependent transcriptional activation of the *IFNB1* promoter (Figs. 2I–L and S2H). These findings demonstrate that although TRIM3 enhances *IFNB1* transcription in NSCLC, excessive IFN-β suppresses TRIM3 expression via its feedback inhibition.

### Reduced TRIM3 expression in NSCLC cells compared with normal cells

**Fig. 3:**
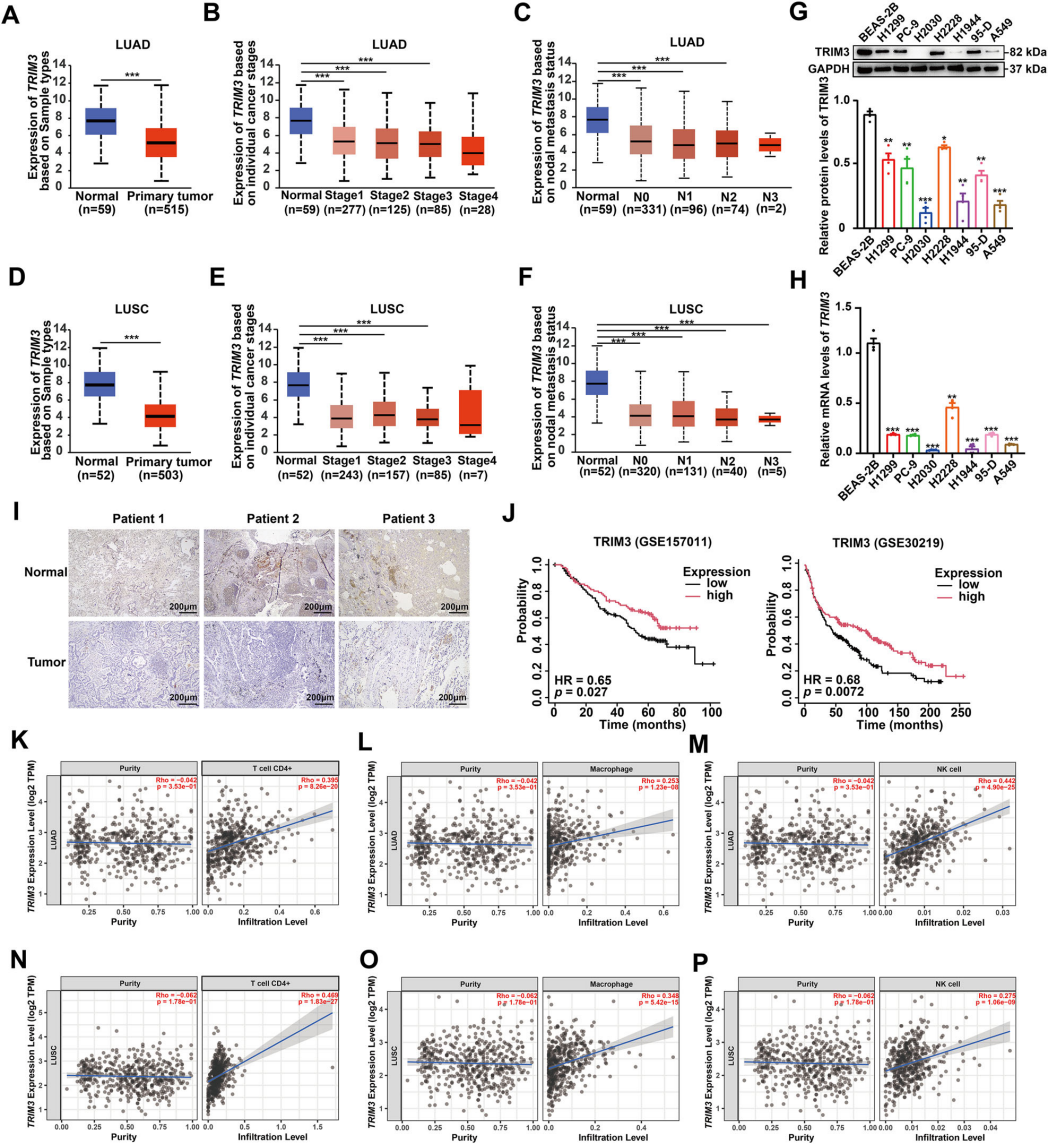
TRIM3 expression is lower in NSCLC cells than in normal cells. **A**–**F** *TRIM3* mRNA levels were analyzed in patients with LUAD and LUSC across paired tumor-normal samples, tumor stages and lymph node metastasis status. **G**, **H** TRIM3 protein and mRNA levels were evaluated in NSCLC cell lines (H1299, PC-9, H2030, H2228, H1944, 95-D and A549) and BEAS-2B cells (n = 4), as assessed by one-way ANOVA. **I** TRIM3 protein levels were analyzed in human NSCLC samples by immunohistochemistry. **J** Kaplan-Meier survival curves were plotted to correlate *TRIM3* mRNA expression with the overall survival of patients with NSCLC in GSE157011 and GSE30219. **K**–**P** The TIMER2.0 database was used to analyze the correlations between *TRIM3* mRNA expression and the infiltration levels of CD4^+^ T cells, NK cells and macrophages in LUAD and LUSC. *p < 0.05; **p < 0.01; ***p < 0.001.

To investigate the role of TRIM3 in NSCLC progression, we first analyzed its mRNA levels in patients with LUAD and LUSC across tumor stages and lymph node metastasis status (N stage). *TRIM3* mRNA expression progressively decreased with increasing tumor stage and N stage in LUAD and LUSC (Fig. 3A–F). WB and qPCR analyses confirmed that TRIM3 expression was lower in lung cancer cells than in normal BEAS-2B bronchial epithelial cells (Fig. 3G, H). Consistent with these findings, immunohistochemistry of human NSCLC tissues revealed lower TRIM3 protein levels in tumors than in adjacent normal tissues (Fig. 3I). Notably, reduced *TRIM3* expression was positively correlated with adverse clinical outcomes in patients with NSCLC (Fig. 3J). Furthermore, *TRIM3* expression correlated positively with the numbers of tumor-infiltrating CD4^+^ T cells, macrophages, and NK cells in LUAD and LUSC, suggesting that TRIM3 plays a role in promoting immune cell recruitment to the tumor microenvironment (Figs. 3K–P and S3). Collectively, these data suggest that TRIM3 plays a tumor-suppressive role in NSCLC progression. Nevertheless, neither overexpression nor knockout of TRIM3 affected the proliferation capability of H1299 and A549 cells, as evidenced by the results of the CCK-8 and colony formation assays (Fig. S4). Taken together, these findings suggest the feedback inhibition on TRIM3 by IFN-β, as mentioned above, likely inhibits the suppressive effect of TRIM3 on NSCLC progression.

### TRIM3 ubiquitinates TLR3 to induce IFN-β secretion

**Fig. 4:**
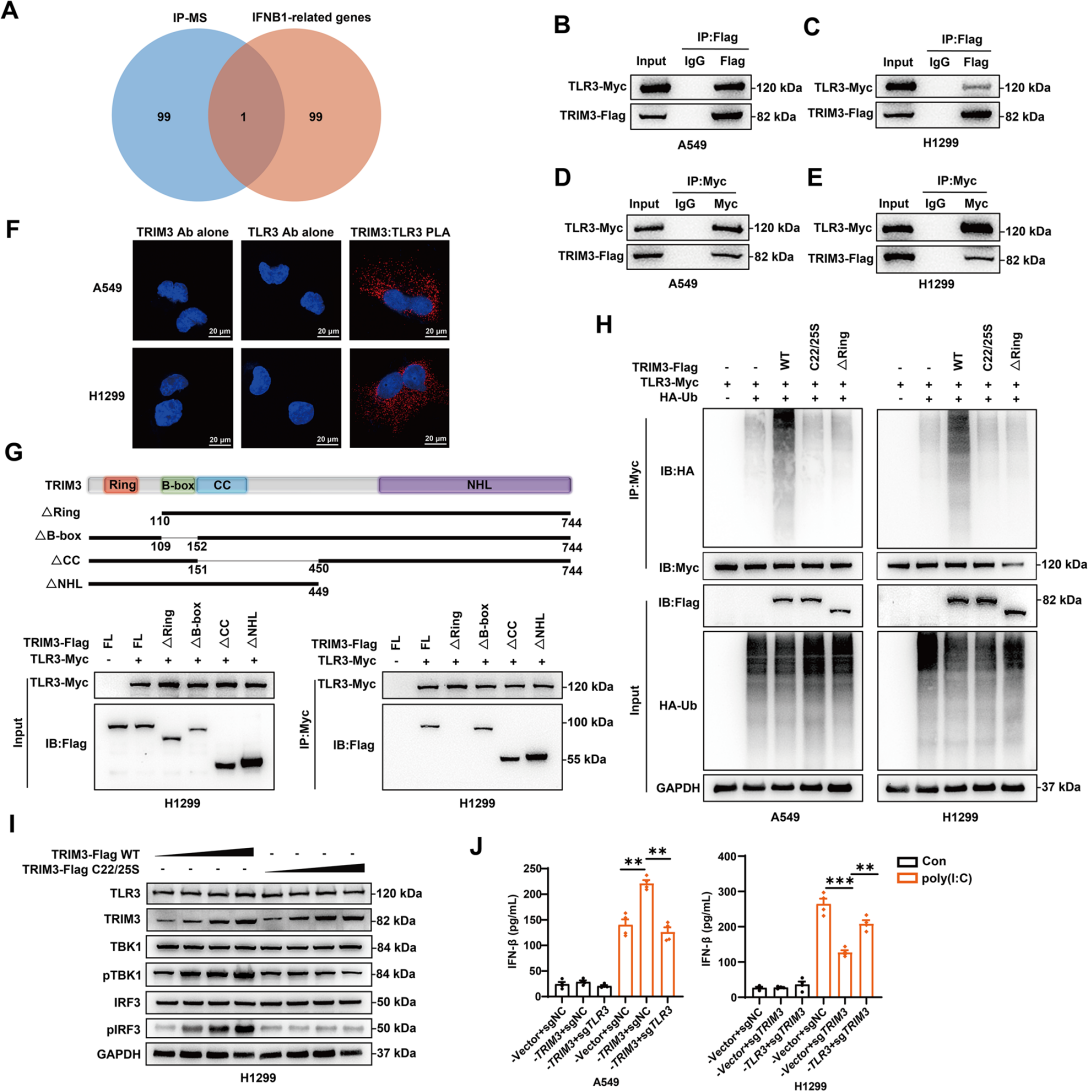
TRIM3 ubiquitinates TLR3 to induce IFN-β secretion. **A** Co-IP coupled with mass spectrometry (IP‒MS) was performed in TRIM3-overexpressing H1299 cells. Immunoprecipitated proteins were quantified by unique peptide counts to identify the top 100 candidates against *IFNB1*-associated proteins. **B**, **C** Co-IP analysis (IP-Flag) validated the physical interaction between TRIM3 and TLR3 in A549 and H1299 cells. **D**, **E** Co-IP analysis (IP-Myc) validated the physical interaction between TRIM3 and TLR3 in A549 and H1299 cells. **F** The interaction between TRIM3 and TLR3 (TRIM3/TLR3; red) was analyzed by PLA in A549 and H1299 cells. **G** Domain mapping of TRIM3 was pictured. Co-IP between TRIM3 deletion mutants (ΔRING, ΔB-box, ΔCC and ΔNHL) and TLR3 was performed in H1299 cells. **H** Ubiquitination assays using HA-ubiquitin and TRIM3 catalytic mutants (C22/25S) were performed in A549 and H1299 cells. **I** WB was performed to detect TBK1 (Ser172) and IRF3 (Ser396) phosphorylation in WT TRIM3 vs. mutant-expressing cells under 20 ng/mL poly(I:C) stimulation. **J** ELISA was performed to quantify TRIM3-dependent IFN-β induction in A549 and H1299 cells (n = 4). Statistical significance was analyzed using one-way ANOVA for multiple comparisons. *p < 0.05; **p < 0.01; ***p < 0.001.

To investigate the mechanisms regulating IFN-β secretion and disrupting feedback inhibition, we performed a coimmunoprecipitation (Co-IP) assay coupled with mass spectrometry analysis following TRIM3 overexpression in H1299 cells, thereby systematically mapping the TRIM3 interactome [29]. Proteins enriched in the immunoprecipitates were ranked by unique peptide counts, and the top 100 candidates were cross-referenced with the top 100 *IFNB1*-associated proteins identified via GeneCards (Fig. 4A). TLR3, a key sensor of dsRNA that triggers IFN-β production and is known to undergo ubiquitin-dependent regulation, emerged as the sole overlapping gene. TLR3 is activated by poly(I:C), a synthetic dsRNA analog [30]. Bidirectional Co-IP assays validated the in vitro physical interaction between TRIM3 and TLR3 in A549 and H1299 cells (Figs. 4B–E and S5). PLA signals (red) confirmed the interaction between TRIM3 and TLR3 in A549 and H1299 cells (Fig. 4F). Like other TRIM family ubiquitin ligases, TRIM3 contains conserved RING, B-box, and coiled-coil domains alongside a variable C-terminal region [20]. Co-IP assays demonstrated that the RING domain is essential for the TRIM3-TLR3 interaction (Fig. 4G). Crucially, compared with wild-type (WT) TRIM3, neither the RING-deleted TRIM3 mutant nor the catalytically inactive mutant (C22/25S) exhibited TLR3 ubiquitination activity (Fig. 4H). Given that TLR3-driven IFN-β production requires TBK1 and IRF3 phosphorylation [31], we also observed concomitant upregulation of phosphorylated TBK1 and IRF3 in the WT TRIM3 groups (but not C22/25S mutant). (Fig. 4I). Complementary qPCR and ELISA analyses in A549 and H1299 cells confirmed that TRIM3 upregulates *IFNB1* transcription and enhances IFN-β secretion under poly(I:C) stimulation. Notably, TLR3 modulation reversed these effects, restoring baseline *IFNB1* mRNA and IFN-β levels (Figs. 4J and S6). Bioinformatic analysis of *TLR3* expression in the NSCLC cohort revealed its progressive downregulation with advanced tumor stage and N stage (Fig. S7A–F). Reduced *TLR3* expression was positively correlated with poor prognosis in patients with NSCLC (Fig. S7G). These results demonstrate that TRIM3 facilitates TLR3 ubiquitination via its RING domain, thereby inducing IFN-β secretion in NSCLC.

### TRIM3 catalyzes the K63-linked ubiquitination of TLR3 at lysine 808

**Fig. 5:**
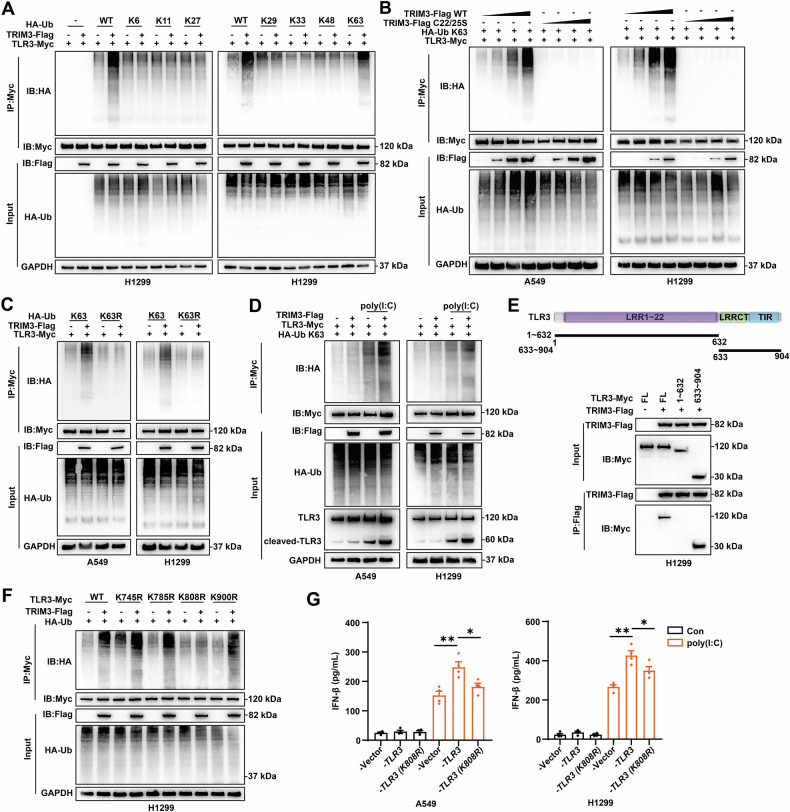
TRIM3 catalyzes the K63-linked ubiquitination of TLR3 at K808. **A** Ubiquitination assays were performed in H1299 cells cotransfected with TRIM3-Flag, TLR3-Myc, and linkage-specific ubiquitin constructs (K6, K11, K27, K33, K48, and K63). **B** Ubiquitination assays were performed to determine the dose-dependent effects of WT and mutant TRIM3 on the K63-linked ubiquitination of TLR3 in A549 and H1299 cells. **C** Ubiquitination assays were performed in A549 and H1299 cells cotransfected with TRIM3-Flag, TLR3-Myc, and linkage-specific ubiquitin constructs (K63, K63R). **D** Ubiquitination assays were performed to examine the enrichment of cleaved TLR3 in A549 and H1299 cells following TRIM3 overexpression and stimulation with or without 20 ng/mL poly(I:C). **E** Domain mapping of TLR3 was pictured. Co-IP between TLR3 deletion mutants (1-632, 633-904) and TRIM3 was performed in H1299 cells. **F** Ubiquitination assays were performed to verify the lysine sites of TLR3 ubiquitinated by TRIM3 in H1299 cells. **G** ELISA was performed to analyze IFN-β secretion levels in A549 and H1299 cells transfected with WT TLR3 or K808R TLR3 (n = 4). Statistical significance was analyzed using one-way ANOVA. *p < 0.05; **p < 0.01.

To elucidate the ubiquitination mechanism, we performed ubiquitination-specific Co-IP assays. Cotransfection with linkage-specific ubiquitin constructs demonstrated that TRIM3 selectively catalyzes the K63-linked ubiquitination of TLR3 (Fig. 5A). In follow-up experiments, we demonstrated that TRIM3 promotes TLR3 ubiquitination through K63-linked (but not K63R mutant) ubiquitination in a dose-dependent manner (Fig. 5B, C). Activation of the TLR3/TBK1/IRF3 signaling axis leading to IFN-β secretion strictly requires the cleavage of TLR3 [32]. Our results also indicated that poly(I:C) stimulation enhanced both TLR3 ubiquitination and cleaved-TLR3 accumulation, and these effects were amplified by TRIM3 overexpression (Fig. 5D). Domain mapping revealed that residues 633–904 in TLR3 were essential for TRIM3 binding (Fig. 5E). Using the GPS-Uber platform, we predicted four candidate lysine ubiquitination sites within this region. Mutation of the TLR3 K808 residue abolished the TRIM3-mediated ubiquitination of TLR3 (Fig. 5F). WT TLR3 significantly increased *IFNB1* mRNA expression and IFN-β secretion, whereas the K808R mutation did not have these effects (Fig. 5G). Taken together, these findings suggest that TRIM3 catalyzes the K63-linked ubiquitination of TLR3 at K808, enabling the activation of IFN-β secretion in NSCLC.

### The TRIM3/TLR3 axis suppresses NSCLC progression

**Fig. 6:**
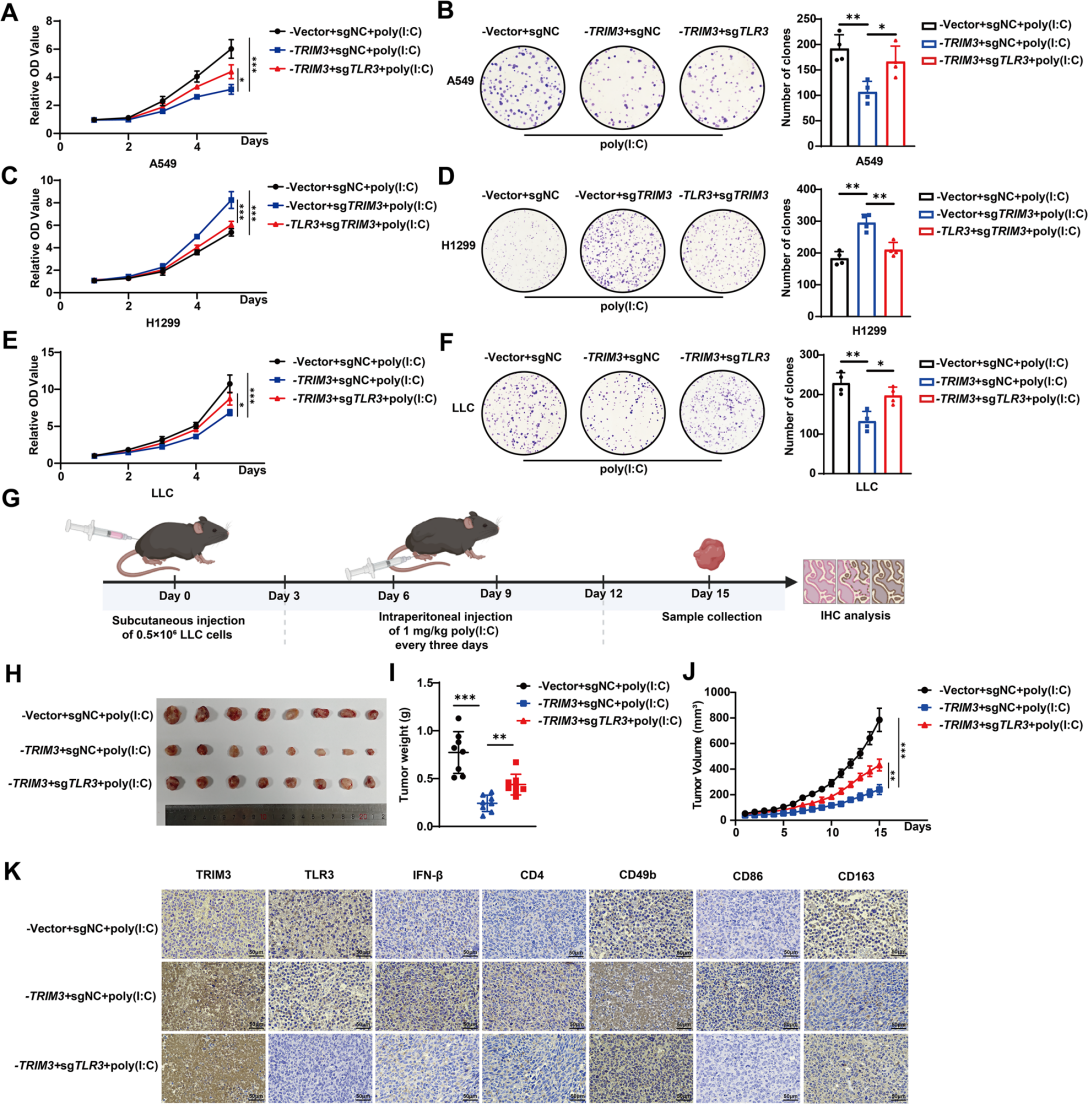
The TRIM3/TLR3 axis suppresses NSCLC progression. **A**–**F** CCK-8 and colony formation assays were performed to validate the effect of the TRIM3/TLR3 axis on A549, H1299 and LLC cell proliferation following 10 ng/mL poly(I:C) stimulation (n = 4). Statistical significance was analyzed using one-way ANOVA. **G** Schematic diagram depicting the experimental workflow of the in vivo experiments. **H**–**J** Syngeneic mouse models were established to evaluate the effect of the TRIM3/TLR3 axis on tumor growth, with intraperitoneal poly(I:C) (1 mg/kg) injections administered every 3 days. Representative images (**H**) and tumor weights (**I**) and volumes (**J**) are shown (n = 8). Statistical significance was analyzed using one-way ANOVA. **K** IHC analysis was performed to evaluate IFN-β, CD4, CD49b, CD86, and CD163 expression in mouse tumors. *p < 0.05; **p < 0.01; ***p < 0.001.

**Fig. 7:**
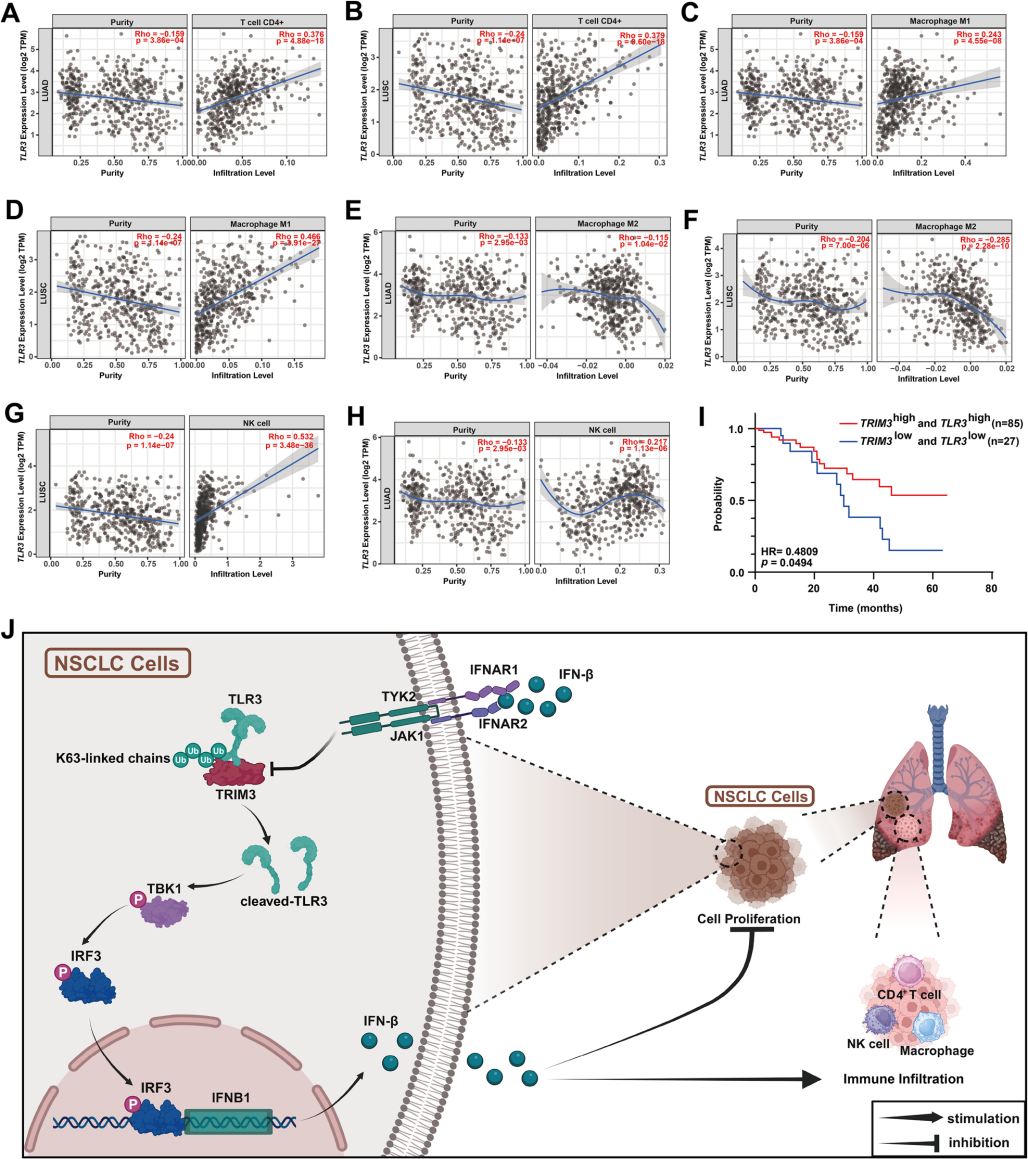
Association of TLR3 with immune infiltration and prognostic value of combined TRIM3/TLR3 expression in NSCLC. **A**–**H** Bioinformatic analysis was conducted to evaluate the correlations between *TLR3* expression and the immune infiltration levels of CD4⁺ T cells, M1 macrophages, M2 macrophages and NK cells in LUAD and LUSC. **I** Kaplan–Meier survival curves were plotted to correlate the combined *TRIM3* and *TLR3* mRNA expression with the overall survival of patients with NSCLC. The log-rank test was used for statistical analysis. **J** Schematic diagram depicting the mechanism by which the TRIM3/TLR3 axis overrides IFN-β feedback inhibition to suppress NSCLC progression.

To investigate the functional role of the TRIM3/TLR3 axis in NSCLC progression, we conducted in vitro and in vivo experiments. Proliferation assays (CCK-8 and colony formation) were performed under poly(I:C) stimulation to activate TLR3 signaling. TRIM3 overexpression suppressed A549 cell proliferation, which was reversed by *TLR3* knockout (Fig. 6A, B). Moreover, *TRIM3* knockout enhanced H1299 proliferation, whereas TLR3 overexpression reversed this effect (Fig. 6C, D). Consistent results were observed in LLC cells (Figs. 6E, F and S8). In our syngeneic mouse models, poly(I:C) was administered intraperitoneally every 3 days. After 15 days, the tumors were excised, weighed and photographed (Fig. 6G). We found that TRIM3 overexpression significantly attenuated tumor growth, but *TLR3* knockout reversed this effect (Fig. 6H–J). Immunohistochemistry of tumor tissues revealed that TRIM3 overexpression increased IFN-β levels and increased the infiltration levels of CD4⁺ T cells, CD49b⁺ NK cells, and CD86⁺ M1 macrophages, but the infiltration levels of CD163⁺ M2 macrophages decreased (Fig. 6K), which is consistent with the results of prior immune infiltration analyses (Fig. 3K–P). Bioinformatic analysis also confirmed the correlations between *TLR3* expression and CD4⁺ T cell, macrophage, NK cell (Fig. 7A–H), CD8^+^ T-cell and dendritic cell infiltration (Fig. S9) in LUAD and LUSC. Clinically, downregulation of *TRIM3* and *TLR3* was positively correlated with poor prognosis in patients with NSCLC (Fig. 7I). Overall, the TRIM3/TLR3 axis suppressed NSCLC progression by directly inhibiting tumor cell proliferation and modulating the infiltration levels of immune cells via IFN-β secretion (Fig. 7J).

## Discussion

IFN-β has garnered significant attention in recent years due to its dual role in suppressing tumor growth and remodeling the immunosuppressive tumor microenvironment [33]. As a prototypic immunotherapeutic agent, IFN-β improves clinical outcomes in malignant tumors [34–36]. However, the therapeutic efficacy of interferon-β is undermined by interference from negative feedback regulatory mechanisms [12]. Our study revealed that TRIM3/TLR3 is involved in a reciprocal negative feedback loop that regulates IFN-β secretion. Mechanistically, TRIM3 promotes TLR3 cleavage via K63 ubiquitination, thereby inducing IFN-β secretion. Both in vitro and in vivo experiments revealed that IFN-β secretion driven by the TRIM3/TLR3 axis suppressed NSCLC cell proliferation and tumor growth. These findings underscore the therapeutic potential of targeting the IFN-β regulatory axis to suppress NSCLC progression, offering novel strategic insights for clinical therapy.

Previous studies have indicated that sustained IFN-β secretion limited by negative feedback regulation could result from the suppression of cytokine signaling (SOCS). SOCS suppresses sustained IFN-β activation by directly inhibiting JAK2 activity, thereby maintaining immune homeostasis [13, 14, 37]. Our study uncovered the TRIM3/TLR3 axis as a previously unknown axis of IFN-β signaling regulation in NSCLC. TRIM3 was found to promote *IFNB1* transcription and expression, while excessive IFN-β suppresses TRIM3 expression via feedback inhibition. Additionally, we found that overexpression of TRIM3 did not inhibit the NSCLC cell proliferation, suggesting the existence of feedback inhibition by IFN-β. Notably, activation of the TRIM3/TLR3 axis drove persistent IFN-β production, thereby overriding the feedback inhibition triggered by excessive IFN-β levels. Our murine studies demonstrated sustained tumor suppression via the TRIM3/TLR3 axis over a 15-day period. Nevertheless, since sustained TRIM3 overexpression is clinically unfeasible, negative feedback regulation may ultimately attenuate IFN-β secretion. Intriguingly, inhibition of TYK2 or knockout of *IFNAR1* and *IFNAR2*, the upstream regulators of the janus kinase-signal transducer and activator of transcription (JAK-STAT) pathway, restored TRIM3 expression. These findings suggest that TRIM3 expression may be suppressed by upstream regulators whose transcription is promoted by STAT signaling. Our future studies will clarify the reciprocal feedback loop between IFN-β and TRIM3 and explore therapeutic strategies targeting key regulators to overcome resistance.

The effects of IFN-β on tumor proliferation are determined by the cellular context. Previous studies have demonstrated that activation of the TBK1/IRF3 pathway restrains NSCLC progression and enhances antitumor immunity [38]. However, the precise mechanisms controlling IRF3-mediated IFN-β secretion remain poorly characterized. Our study revealed that the TRIM3/TLR3 axis mediates TBK1/IRF3 pathway activation and drives IFN-β secretion, conferring antiproliferative effects against NSCLC. IFN-β inhibits tumor cell proliferation and survival while mobilizing systemic immune responses through enhanced M1-polarized macrophage phenotype, NK cell proliferation, and T-cell effector function [39–41]. Notably, our bioinformatic and in vivo data demonstrated that TRIM3 and TLR3 expression increased the infiltration levels of CD4⁺ T cells, M1 macrophages, and NK cells but decreased the infiltration level of M2 macrophages. These results indicate that compromised TRIM3/TLR3 signaling shapes an immunosuppressive tumor microenvironment that facilitates tumor immune evasion. In future studies, the functional mechanisms through which TRIM3-induced IFN-β affects tumor immune cells remain to be fully elucidated.

TRIM3, a RING domain-containing E3 ubiquitin ligase initially characterized for its ability to regulate intracellular trafficking [42], has recently been shown to have tumor-suppressive effects across multiple cancers [43, 44]. Here, we demonstrated that TRIM3 expression is significantly downregulated and is correlated with prognosis in patients with NSCLC. This study revealed that TRIM3 catalyzes the K63-linked ubiquitination of TLR3 at K808 via its RING domain, inducing IFN-β secretion to suppress NSCLC progression. These findings expand the known immunomodulatory roles of TRIM family proteins and highlight the catalytic necessity of the Cys22/Cys25 residues in TRIM3, providing a structural basis for the development of small-molecule agonists targeting TRIM3.

While this study elucidated the role of the TRIM3/TLR3 axis in NSCLC suppression, the mechanistic details of its immunomodulatory effects, including its regulation of CD4⁺ T-cell priming, NK cell cytotoxicity, and macrophage polarization, remain to be fully elucidated. Additionally, the limited clinical cohort size warrants validation in expanded cohorts of patients treated with immunotherapy. Finally, we aimed to elucidate JAK-STAT downstream effectors that repress TRIM3 expression, and pharmacological targeting holds promise for overcoming IFN-β therapeutic resistance.

## Conclusion

In summary, our study reveals a reciprocal feedback loop governing IFN-β secretion in NSCLC. TRIM3 promotes *IFNB1* expression, whereas excessive IFN-β induces TRIM3 downregulation via feedback inhibition. Notably, the TRIM3/TLR3 axis overrides this feedback inhibition through TRIM3-mediated K63-linked ubiquitination of TLR3 at K808, thereby suppressing NSCLC cell proliferation and inhibiting tumor growth. These findings deepen mechanistic insights into IFN-β signaling dynamics and identify the TRIM3/TLR3 axis as a promising therapeutic target in NSCLC.

## Supplementary information

Uncropped Western blot images

Supplementary Figures

Supplementary Tables

## Data Availability

The mass spectrometry proteomics data have been deposited to the ProteomeXchange Consortium (https://proteomecentral.proteomexchange.org) via the iProX partner repository with the dataset identifier PXD068239. Other datasets used and analyzed in this study are available from the corresponding author.
