# Supplementary material for: The TRIM3/TLR3 axis overrides IFN-β feedback inhibition to suppress NSCLC progression
Source: Cell Death Dis. 2026 Jan 16;17(1):44. doi: 10.1038/s41419-025-08265-w (PMC12811290; doi:10.1038/s41419-025-08265-w)
Supplement: Supplementary file 1 — Uncropped Western blot images [file 41419_2025_8265_MOESM1_ESM.docx]

**
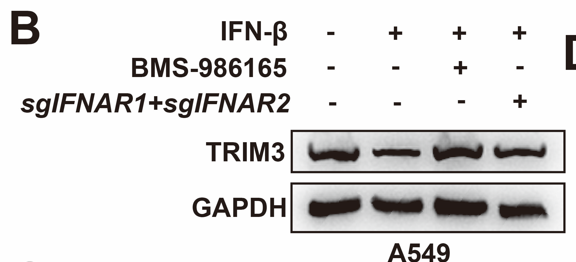
Fig. 2B**


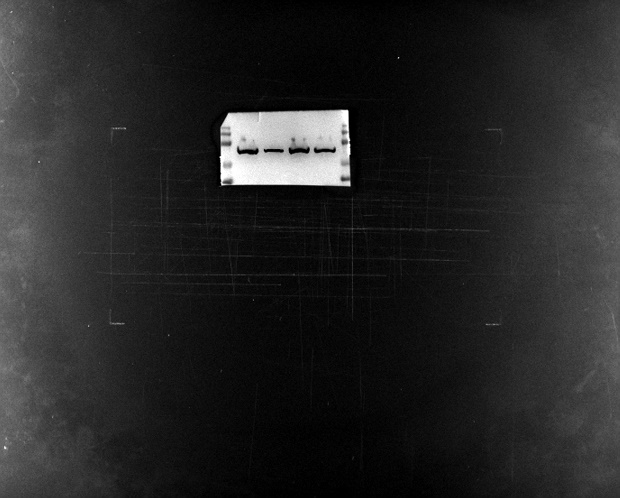
**TRIM3:**


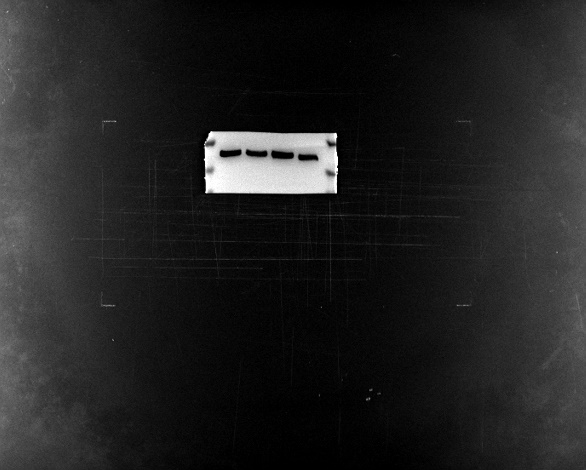
**GAPDH:**

**
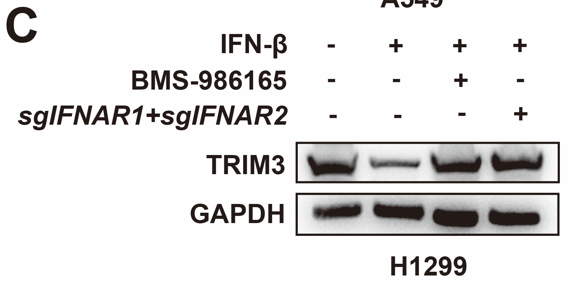
Fig. 2C**


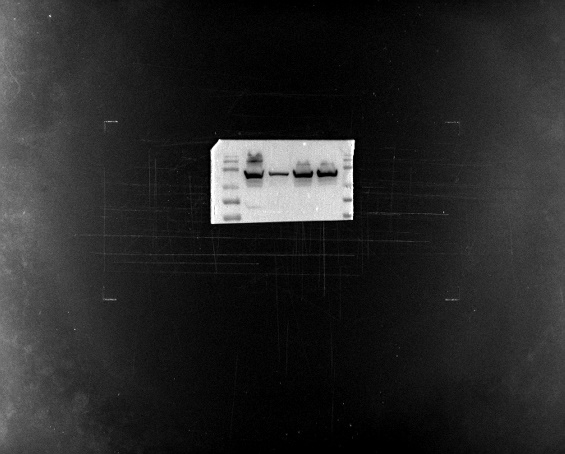
**TRIM3:**


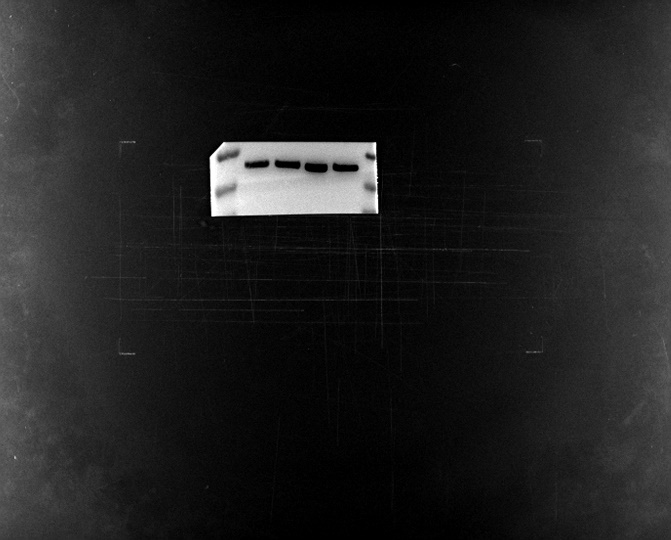
**GAPDH:**

**
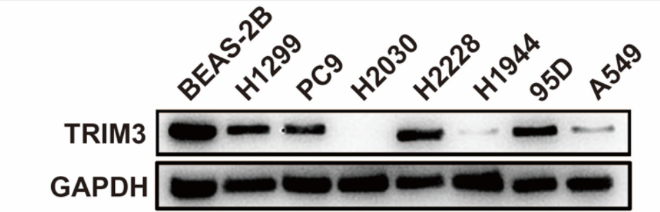
Fig. 3G**


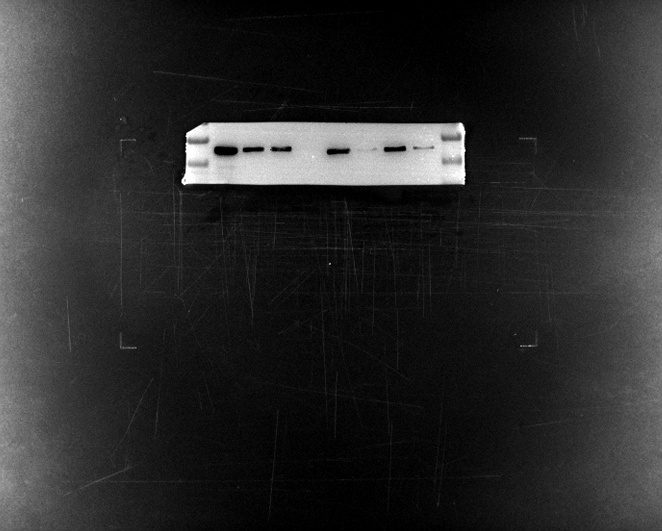

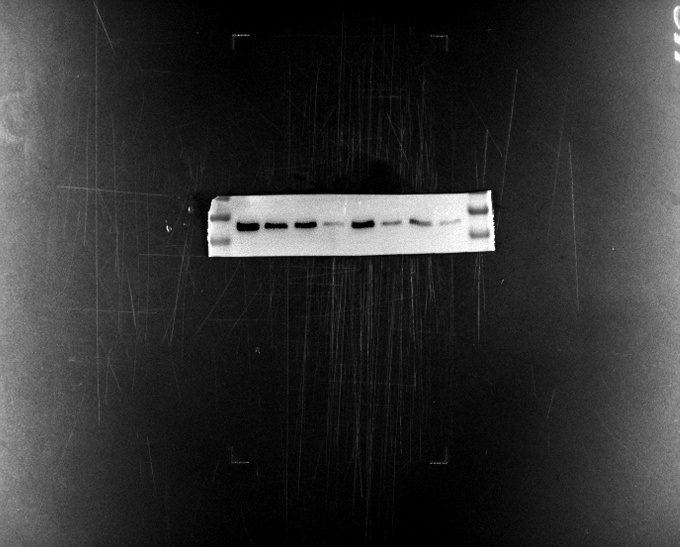

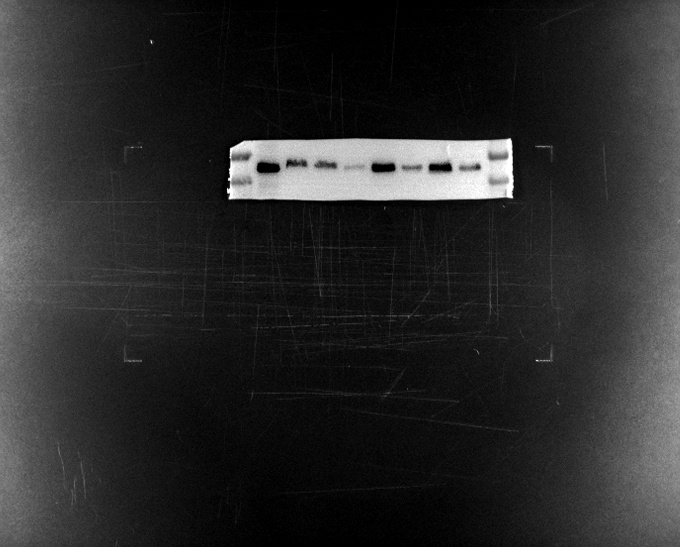

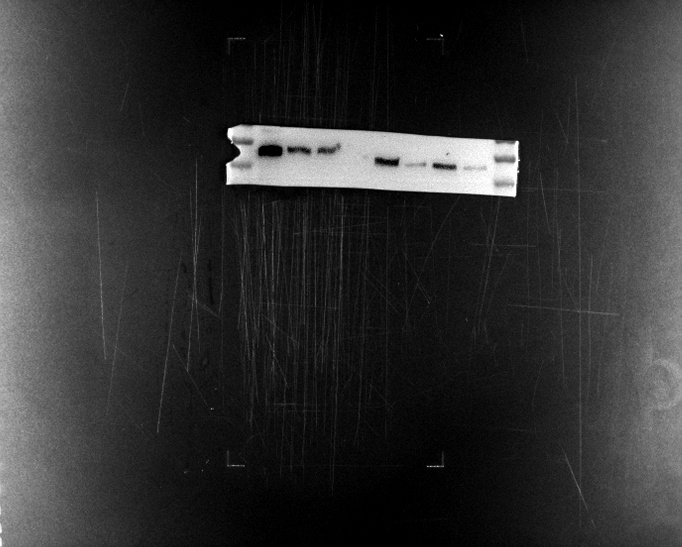
**TRIM3:**


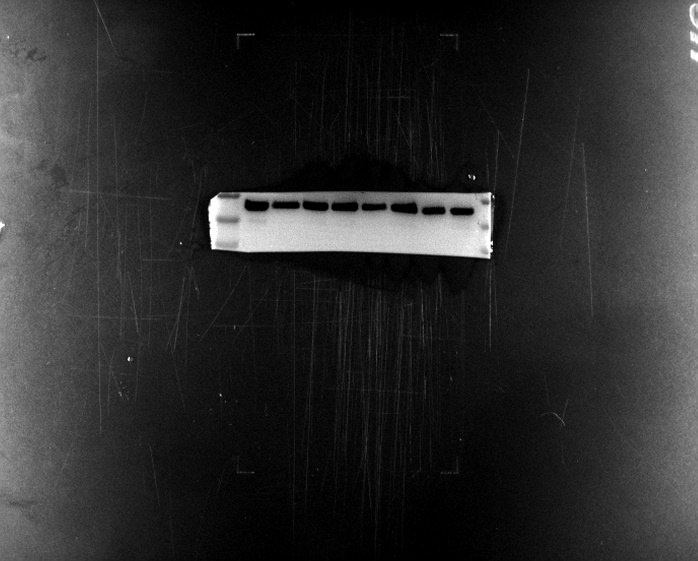

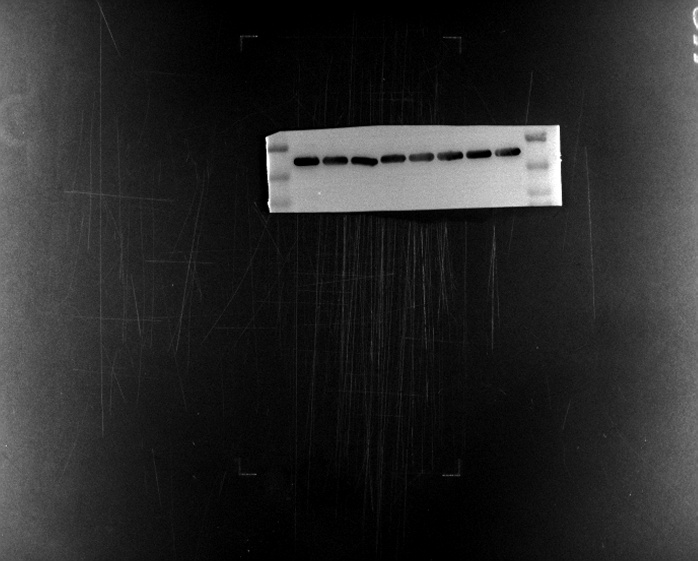

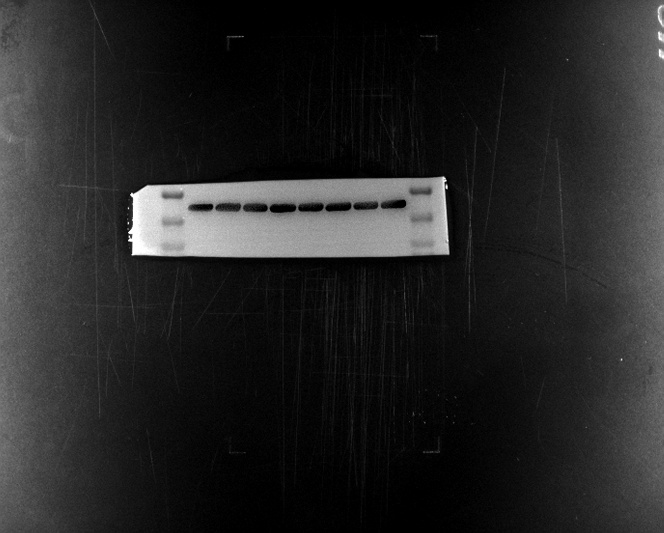
**
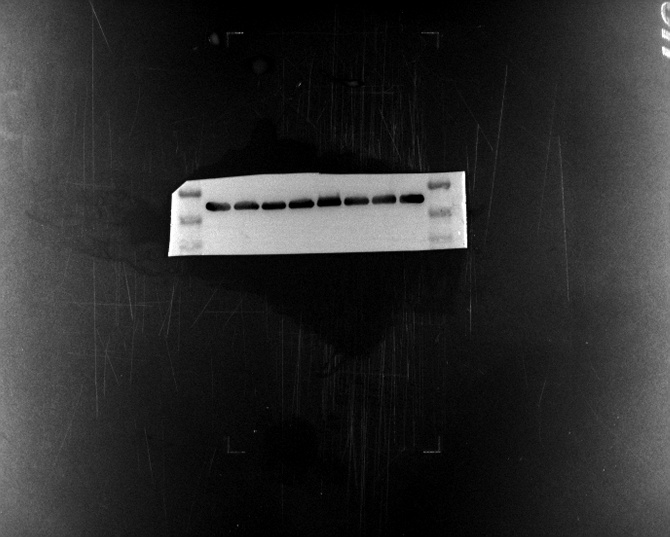
GAPDH:**

**
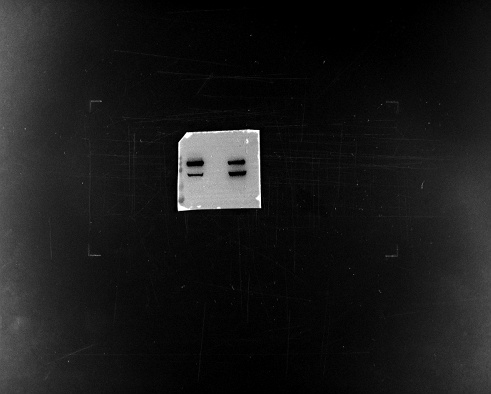

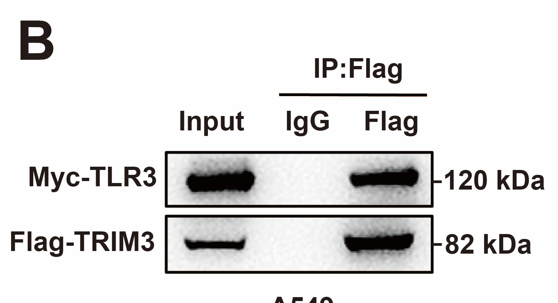
Fig. 4B**

**
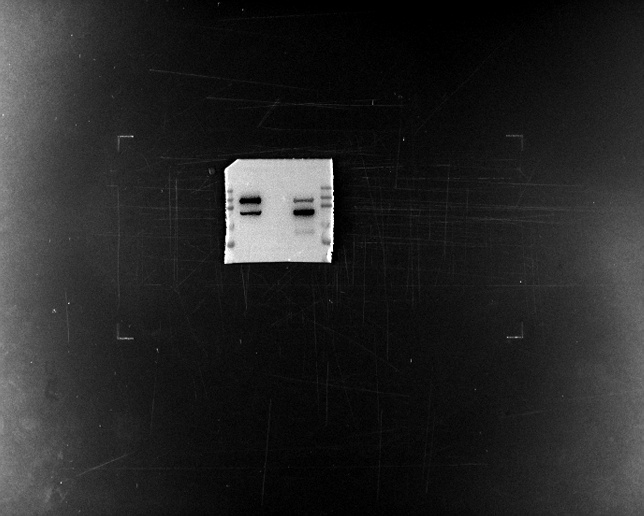

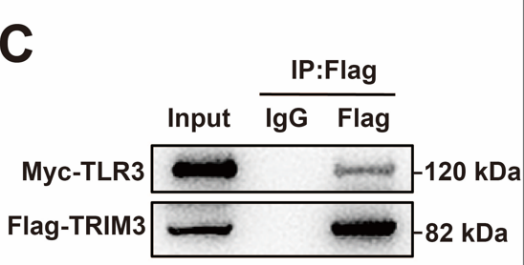
Fig. 4C**

**
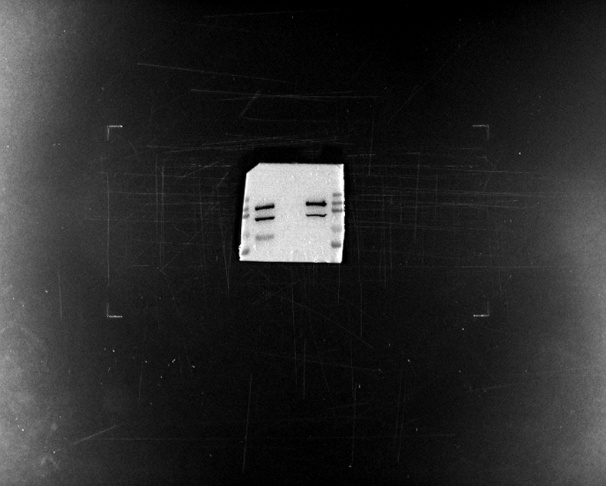

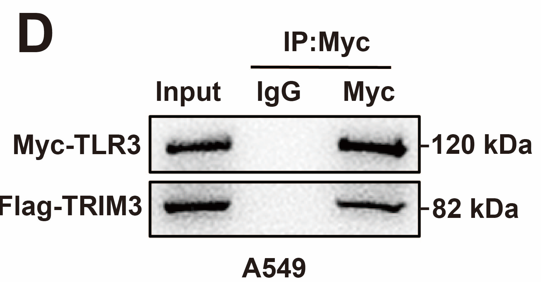
Fig. 4D**

**
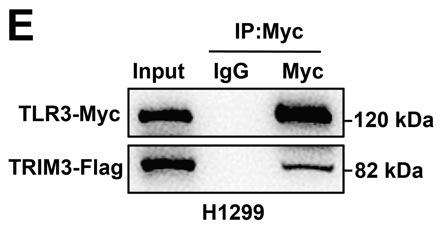
Fig. 4E**

**
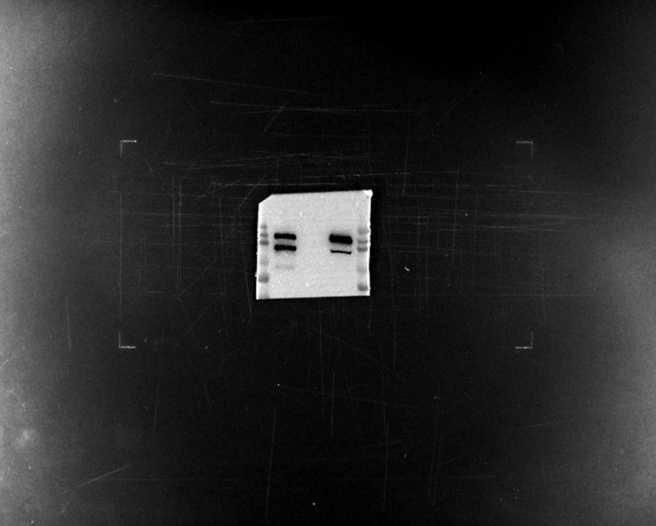
**

**
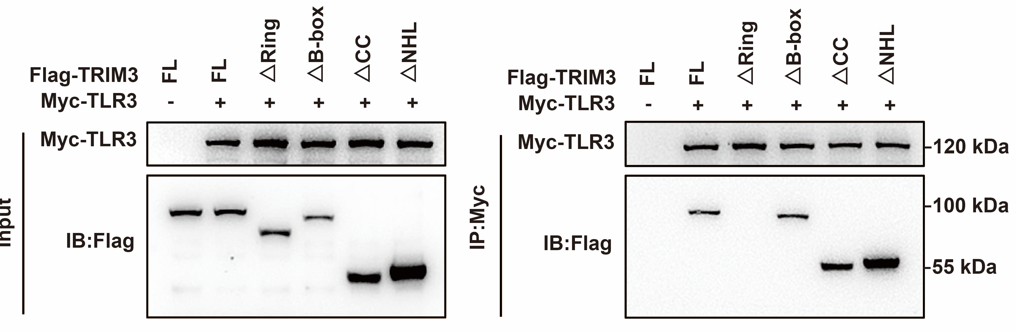
Fig. 4G**

**
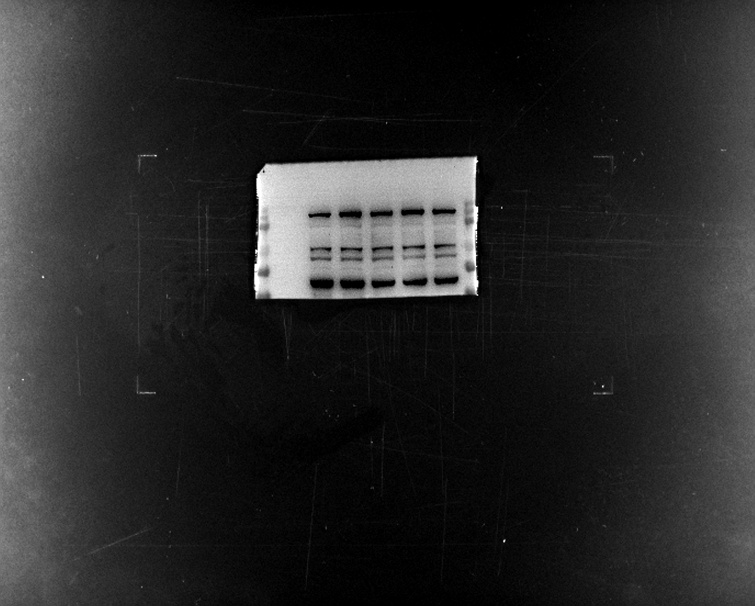
Input-Myc**

**
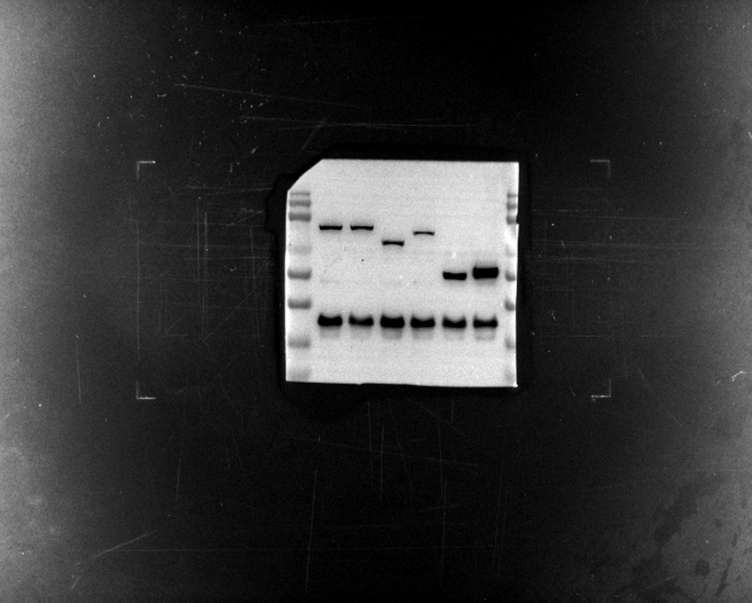
Input-Flag:**

**
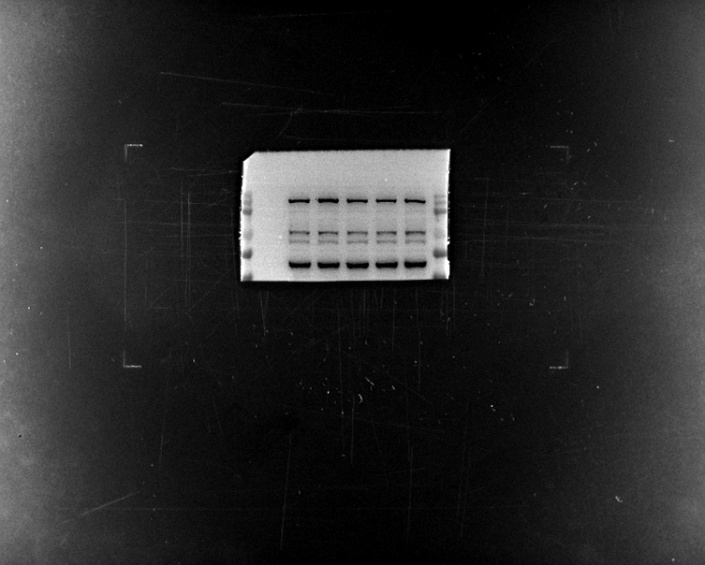
IP-Myc:**

**
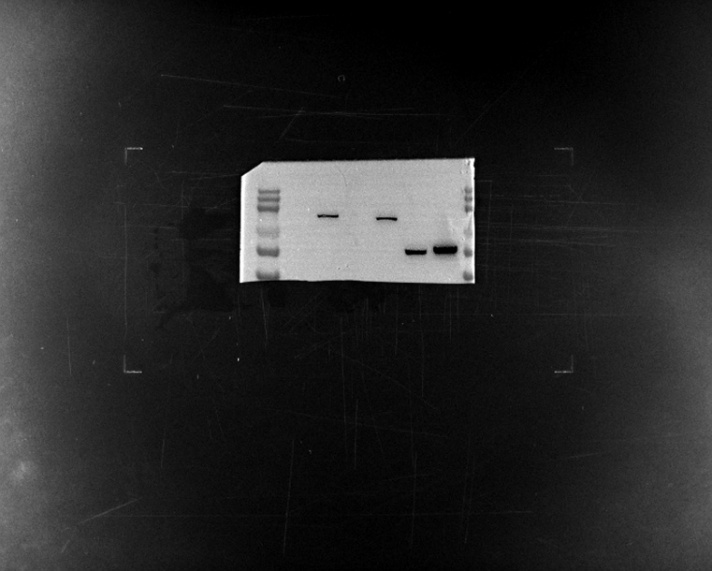
IP-flag:**

**
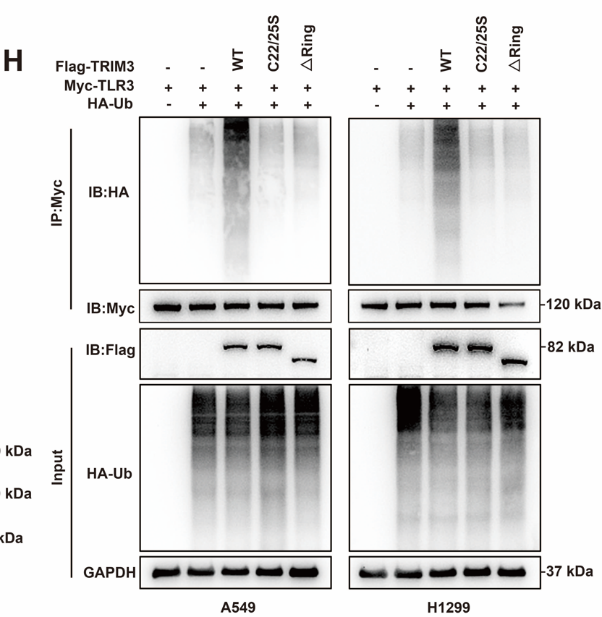
Fig. 4H**

**
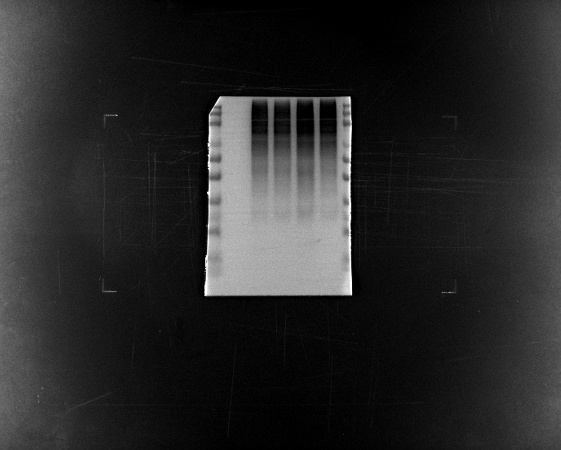
A549-Input-UB:**

**
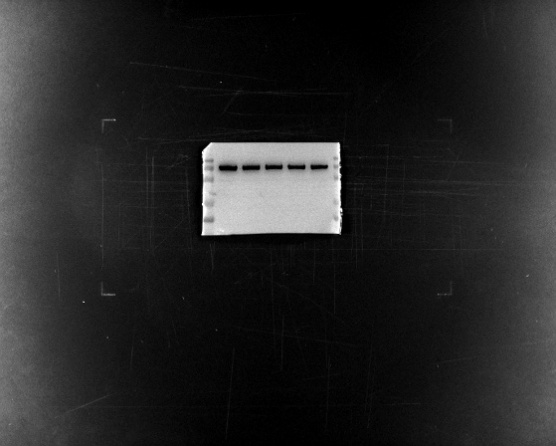
A549-IP-Myc:**

**
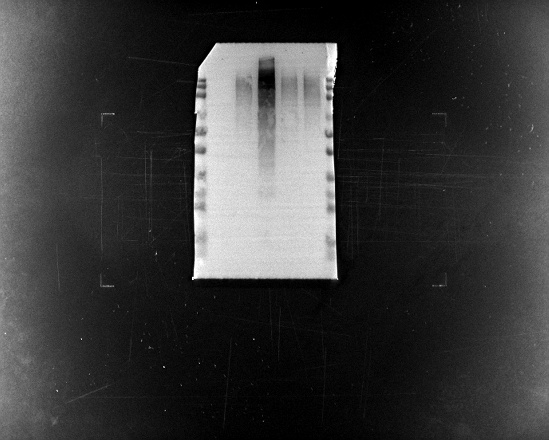
A549-IP-HA:**

**
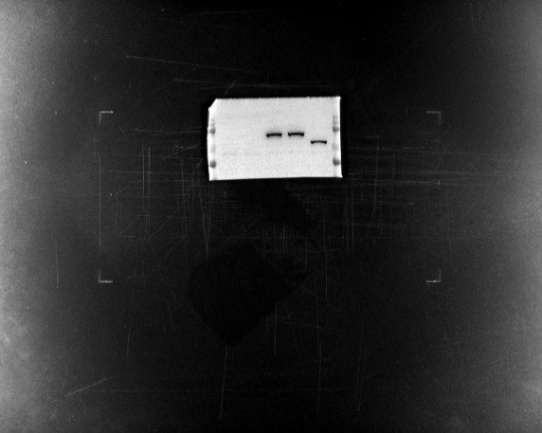
A549-Input-flag:**

**
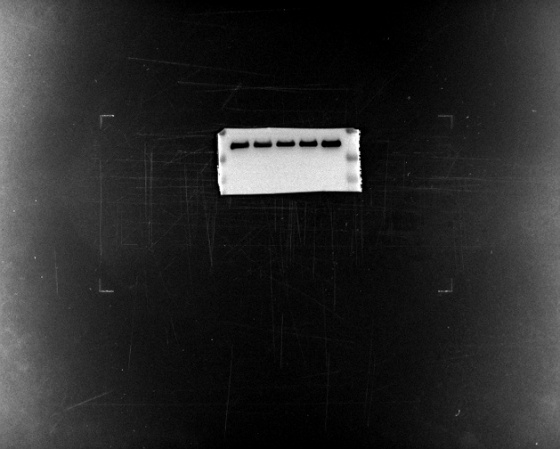
A549-Input-GAPDH:**

**
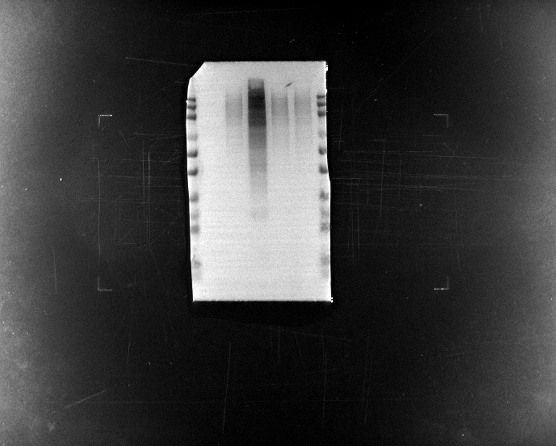
H1299-IP-UB:**

**
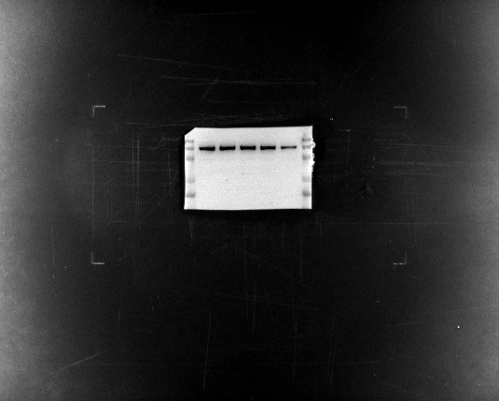
H1299-IP-Myc:**

**
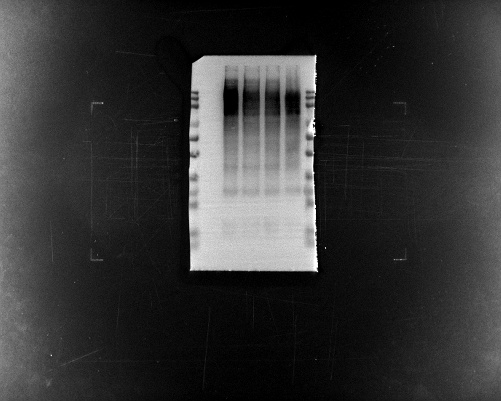
H1299-Input-UB:**

**
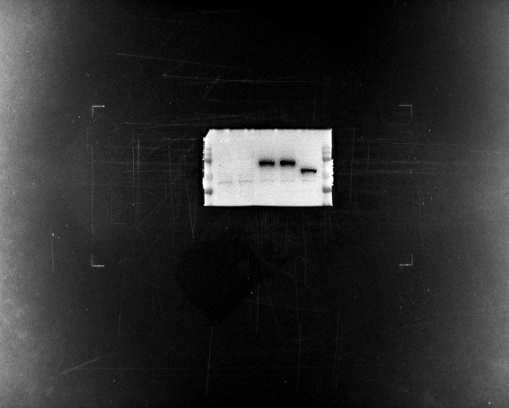
H1299-Input-flag:**

**
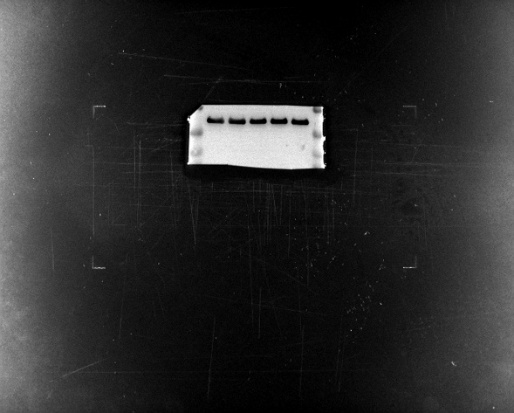
H1299-Input-GAPDH:**

**
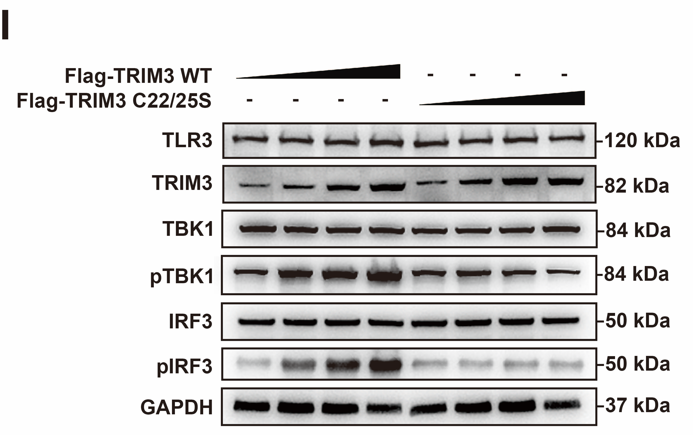
Fig. 4I**

**
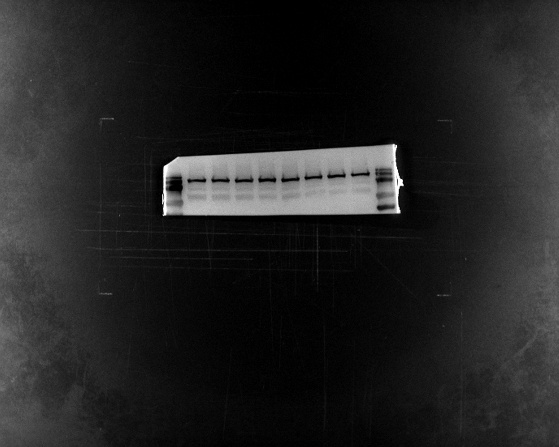
TLR3:**

**
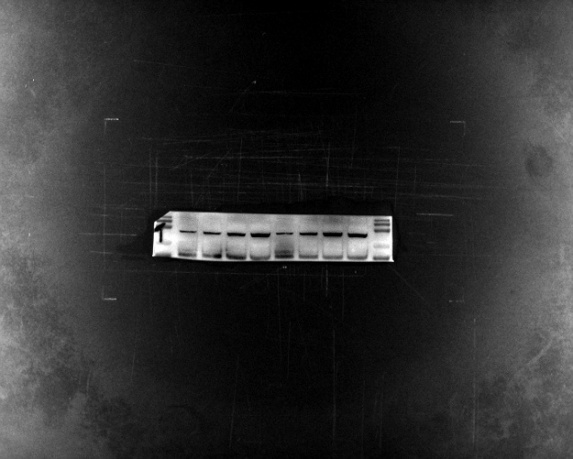
TRIM3:**

**TBK1:**


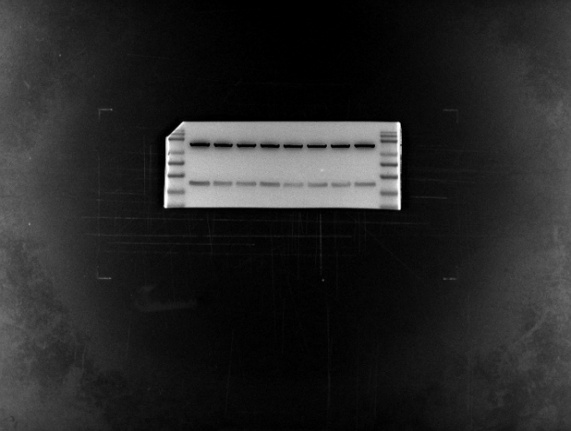


**
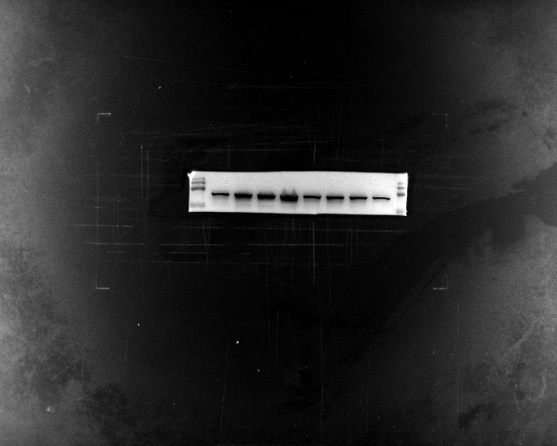
pTBK1:**

**
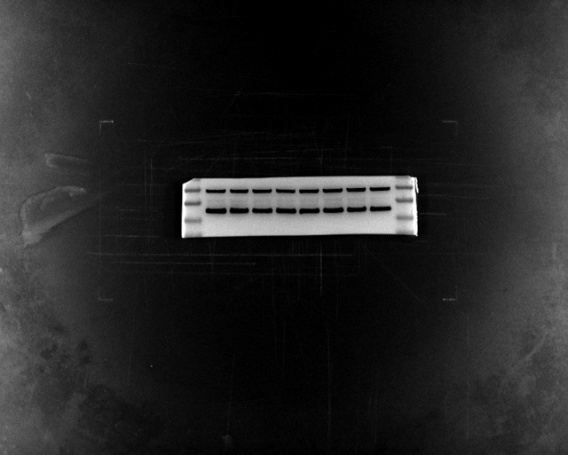
IRF3:**

**
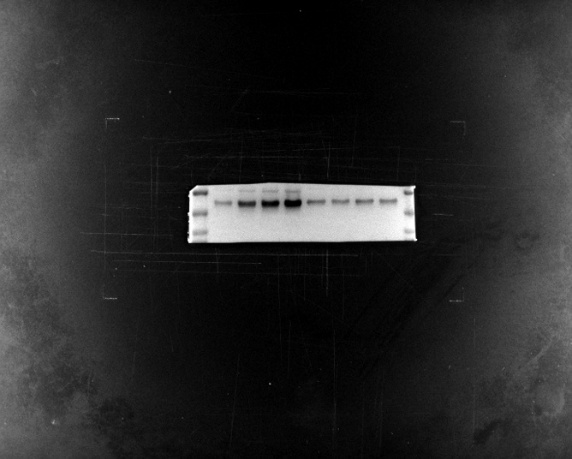
pIRF3:**

**
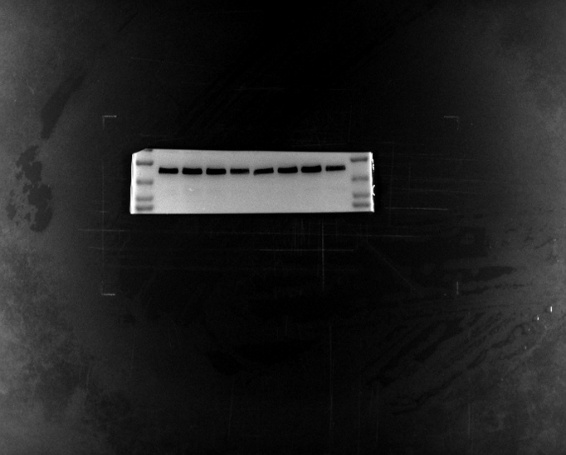
GAPDH:**

**
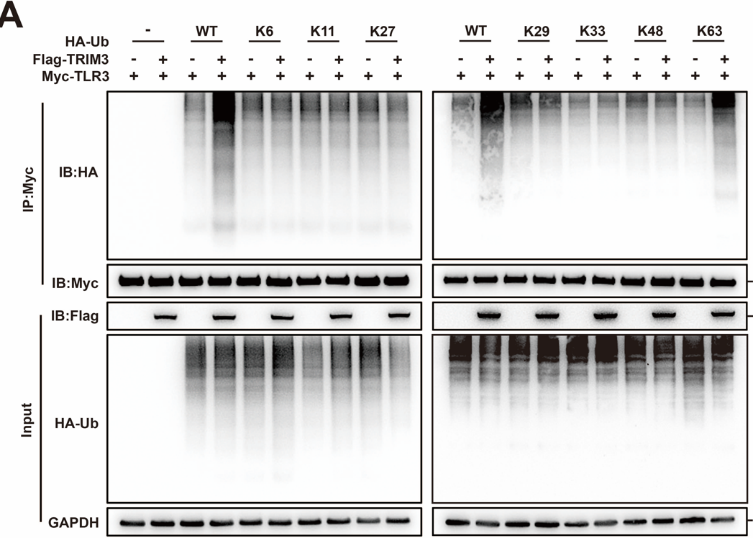
Fig. 5A**

**
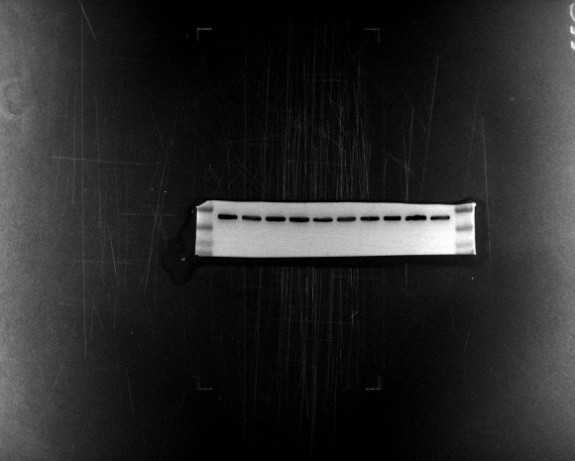

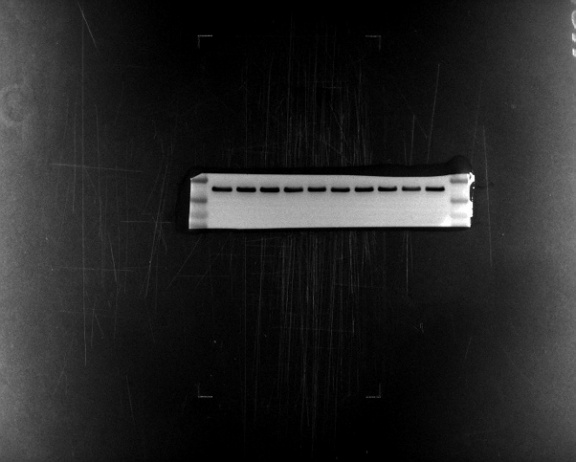
GAPDH:**

**
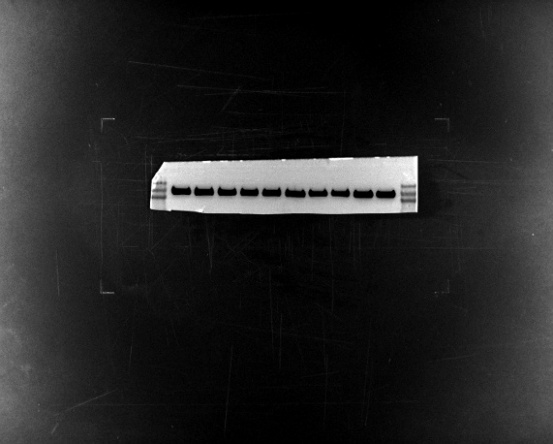

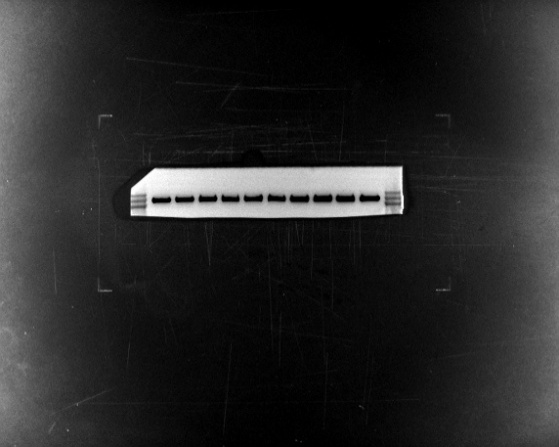
IP-Myc:**

**
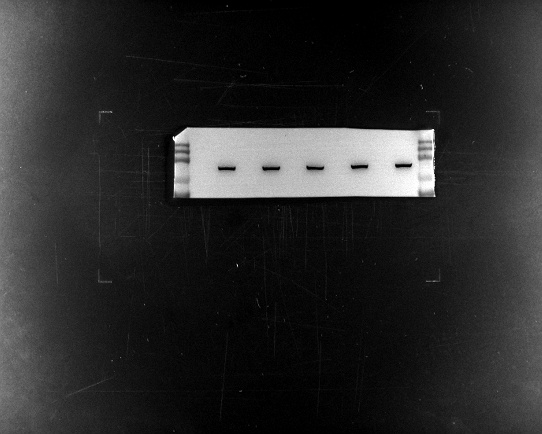

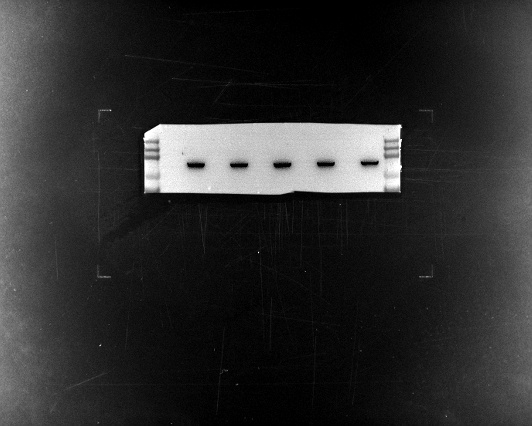
Input-Flag:**

**
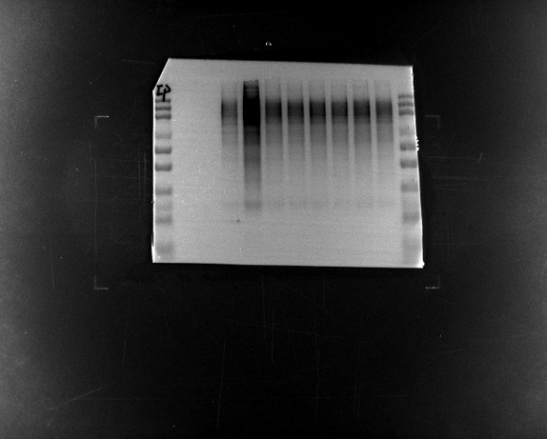

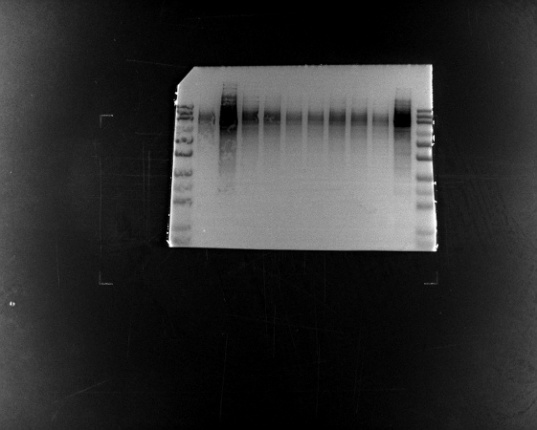
IP-UB:**

**
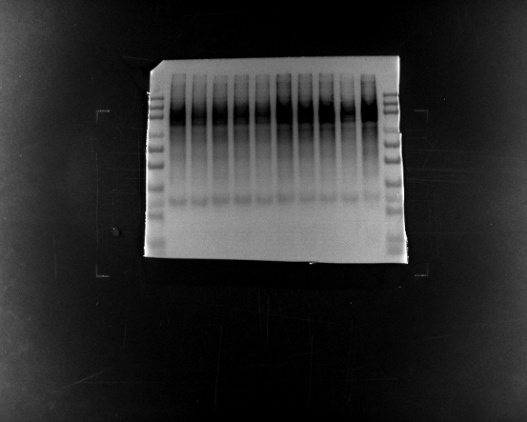

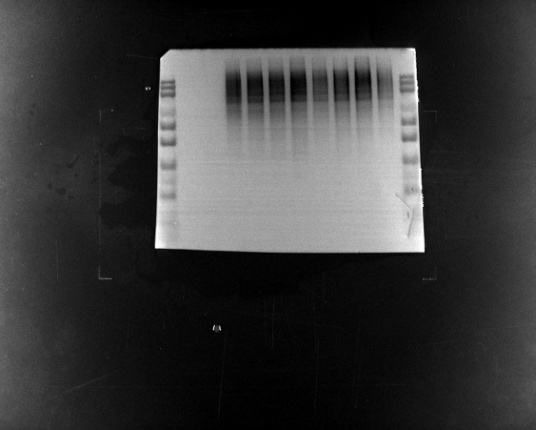
Input-UB:**

**
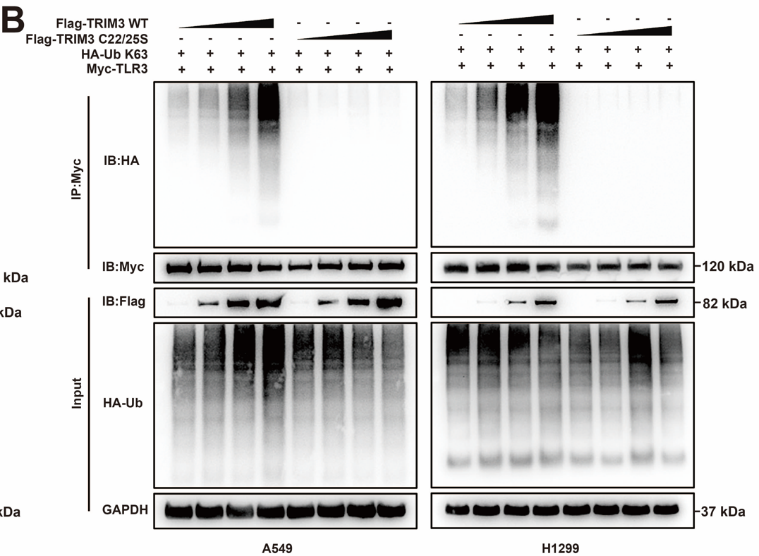
Fig. 5B**

**
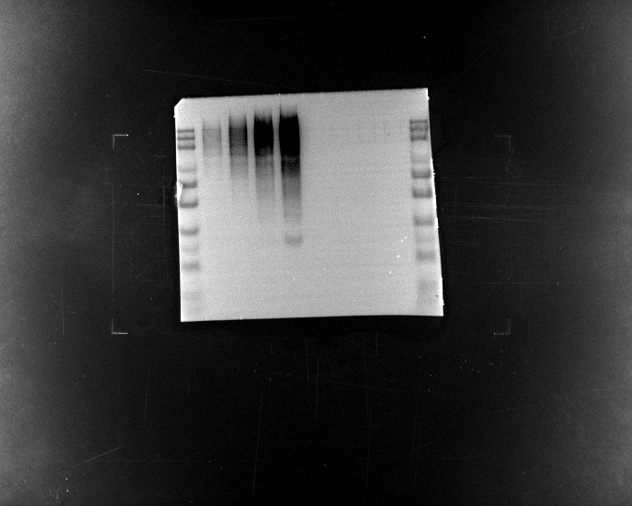

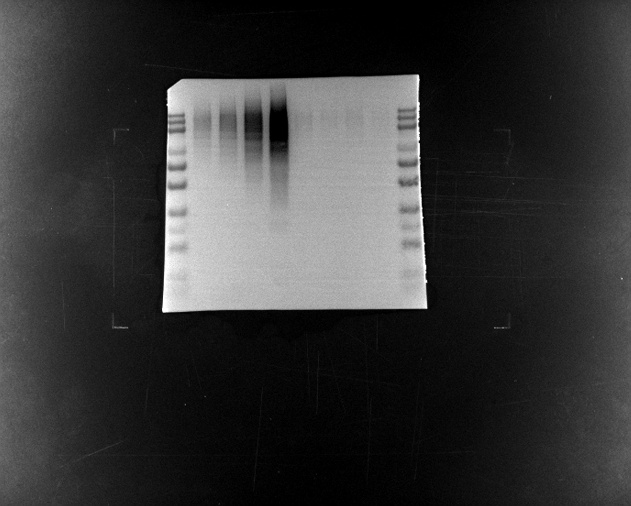
IP-UB:**

**
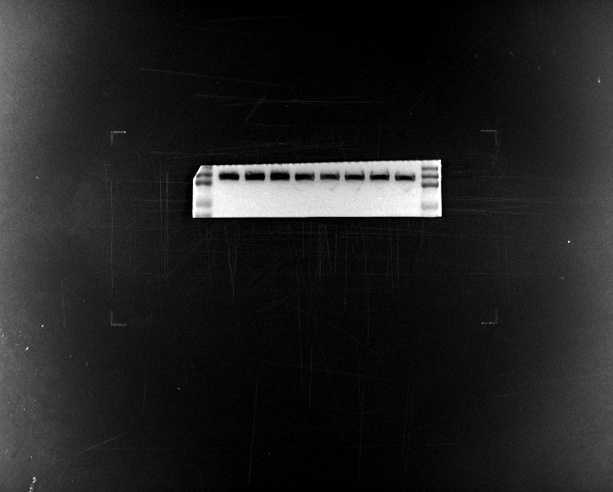

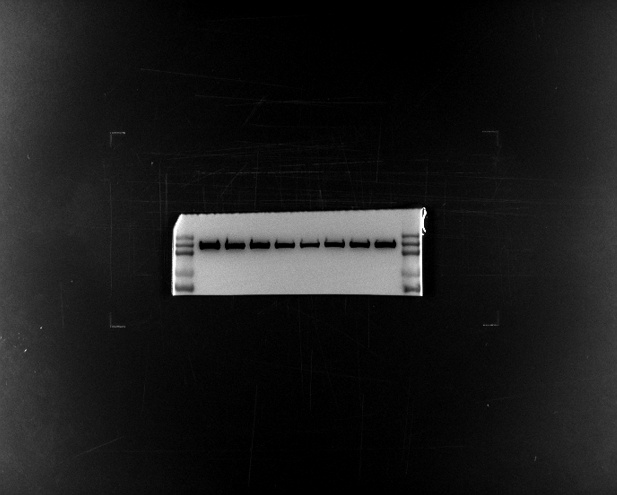
IP-Myc:**

**
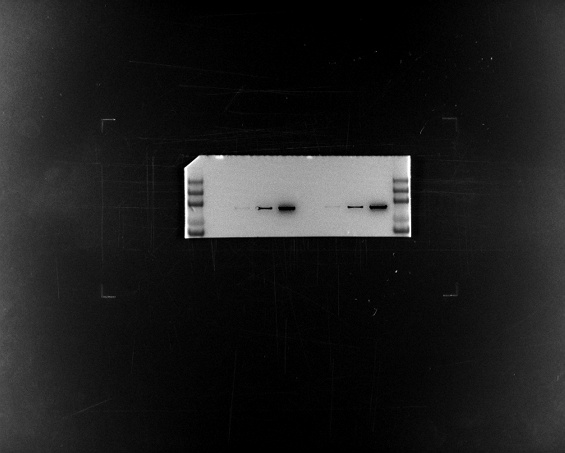

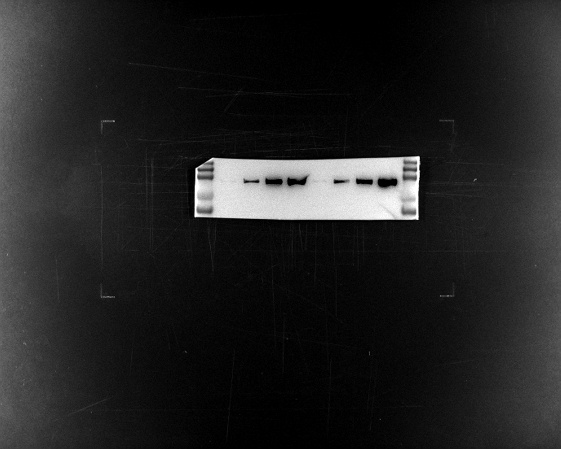
Input-Flag:**

**
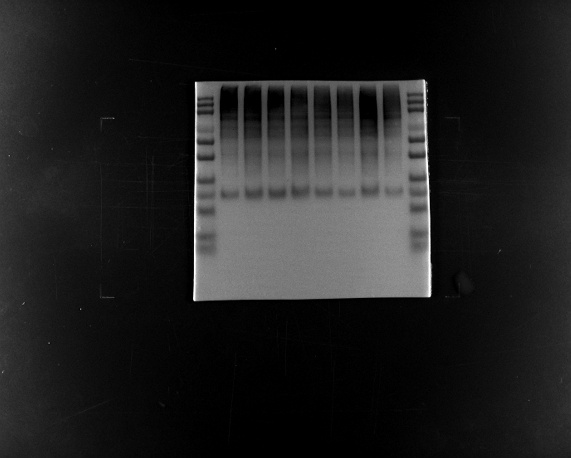

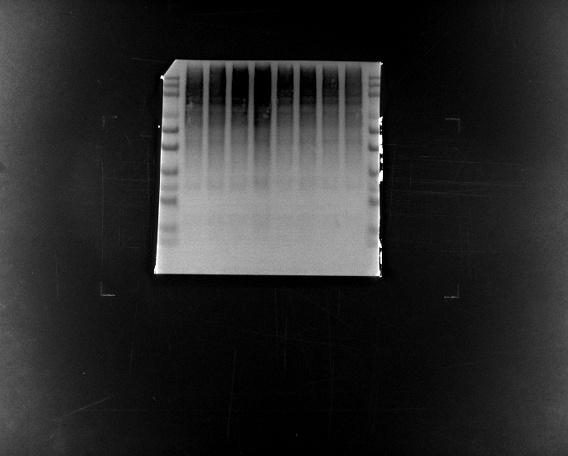
Input-UB:**

**
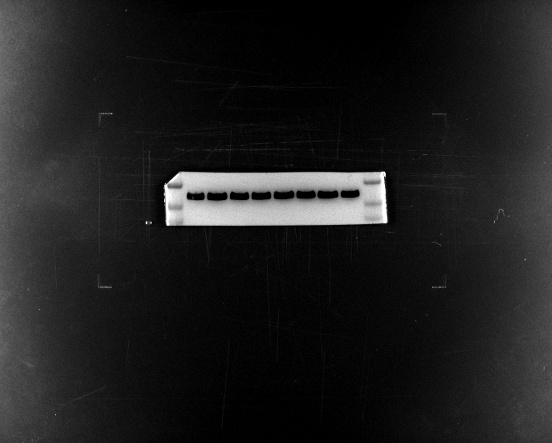

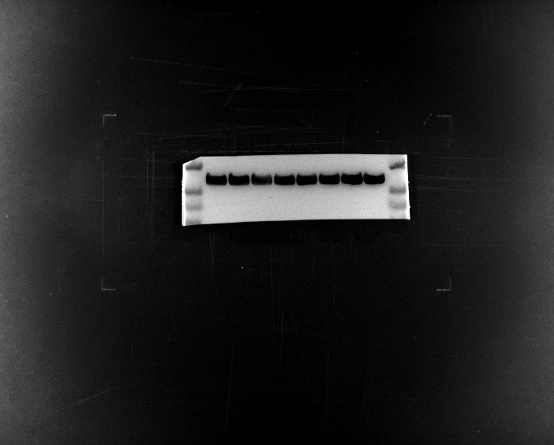
GAPDH:**

**
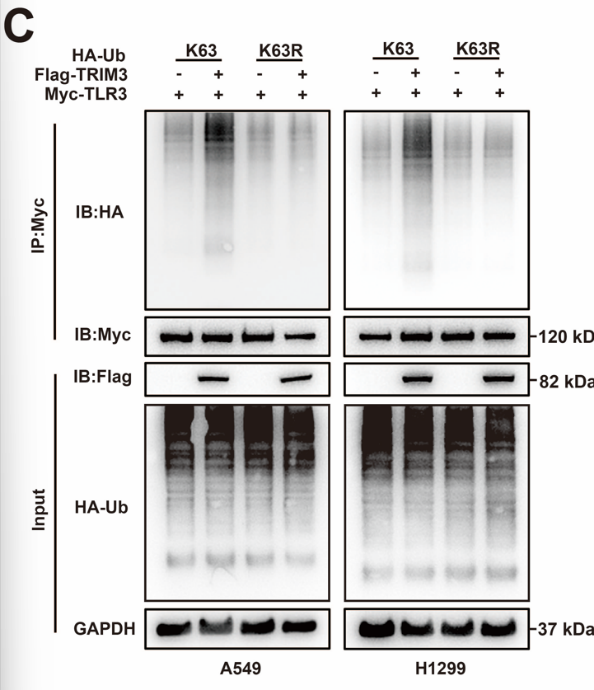
Fig. 5C**

**
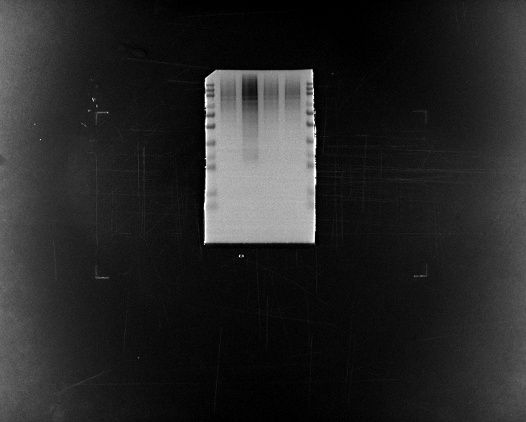

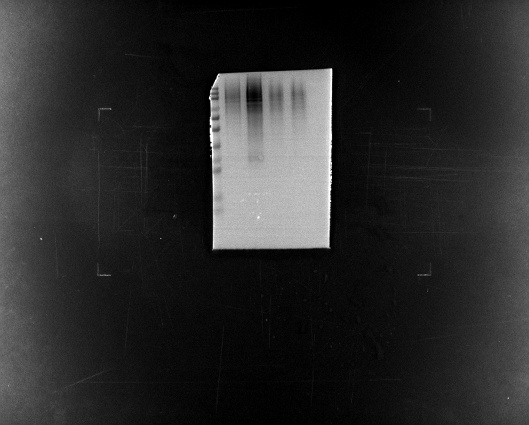
IP-UB:**

**
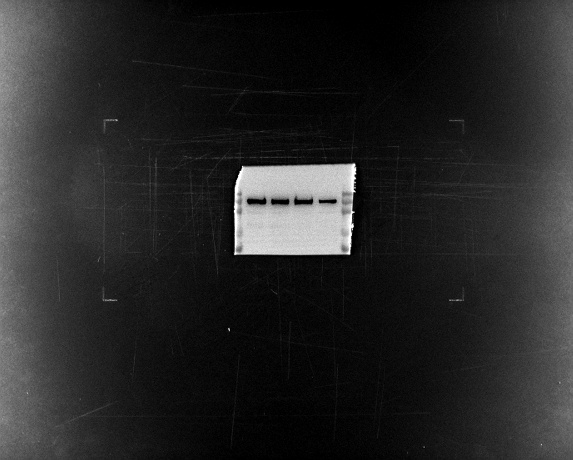

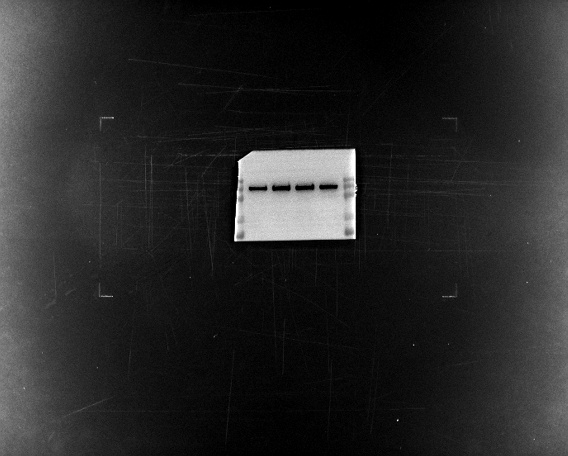
IP-Myc:**

**
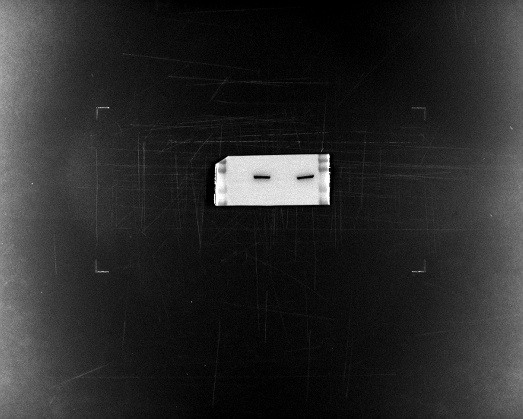

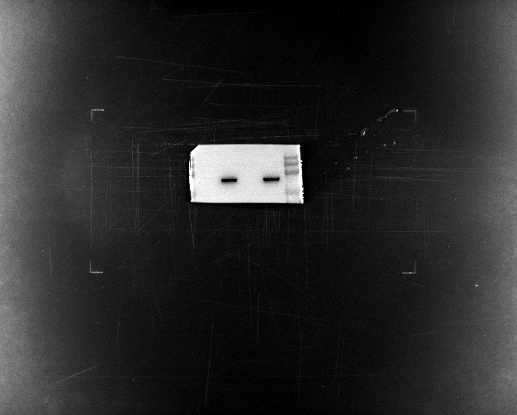
Input-flag:**

**
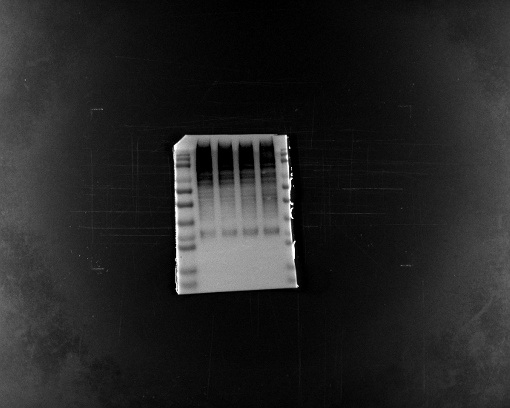

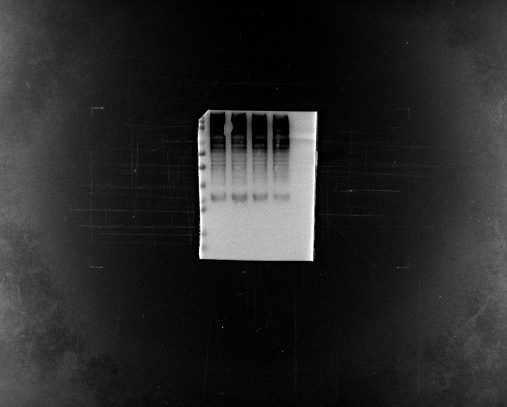
Input-UB:**

**
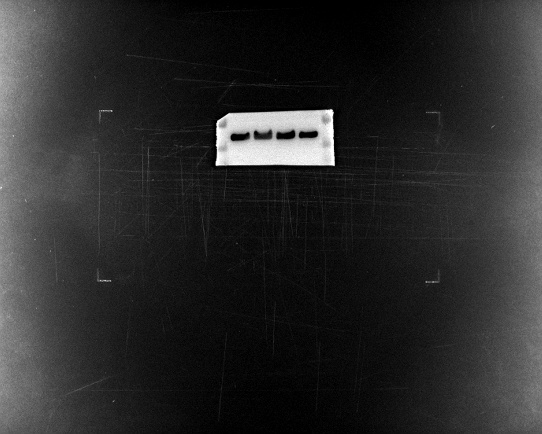

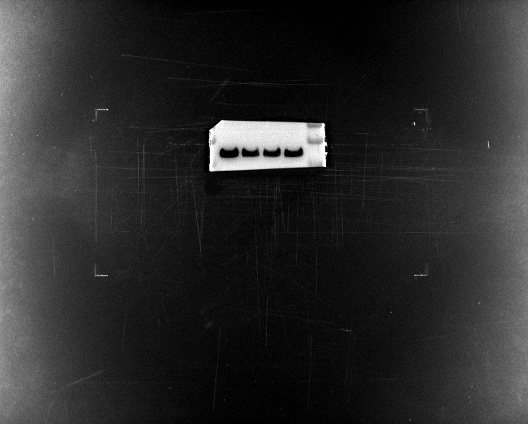
GAPDH：**

**
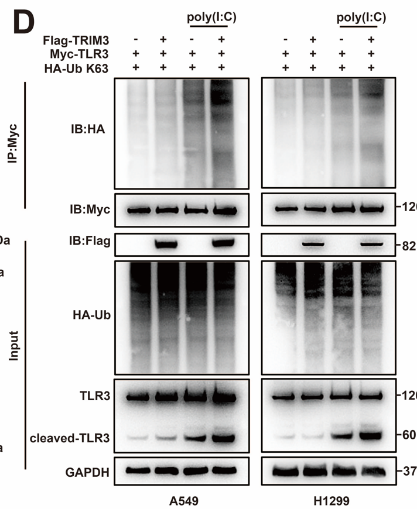
Fig. 5D**

**
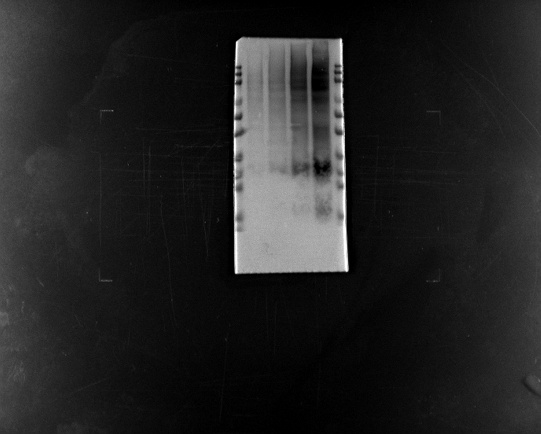

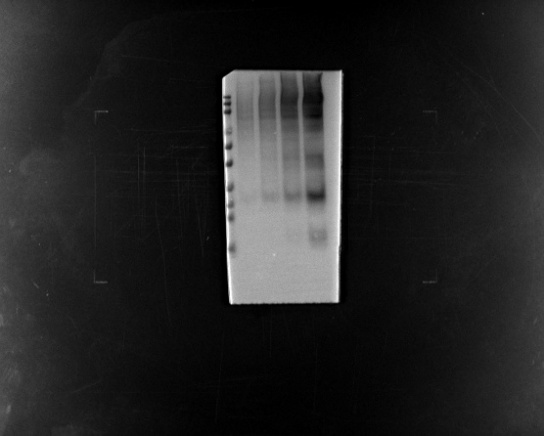
IP-UB:**

**
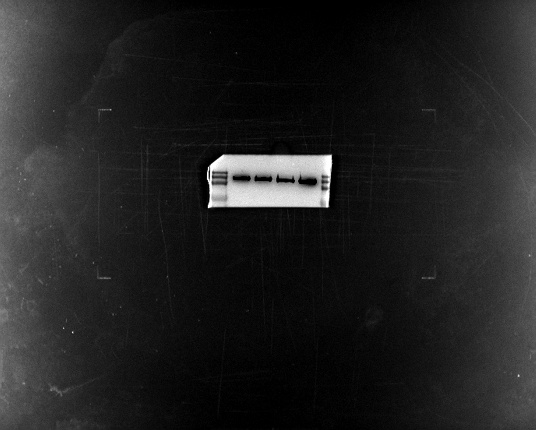

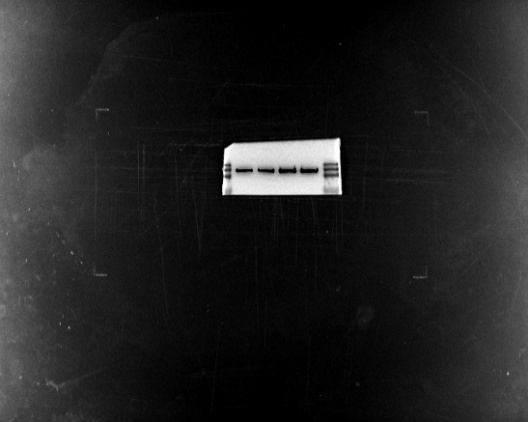
IP-Myc:**

**
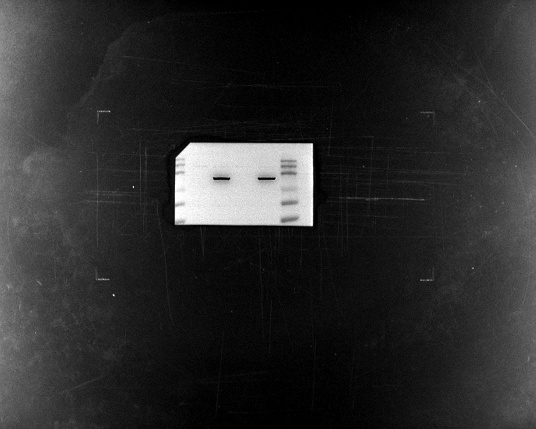

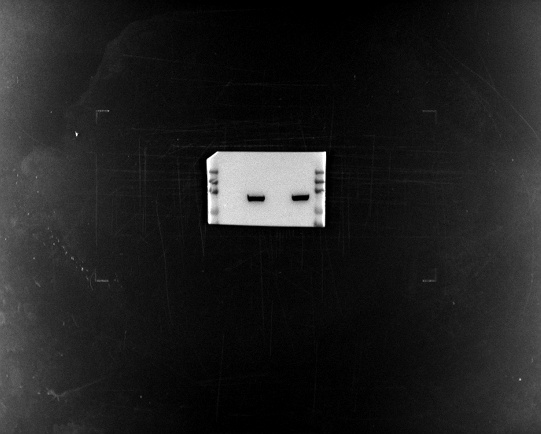
Input-flag:**

**
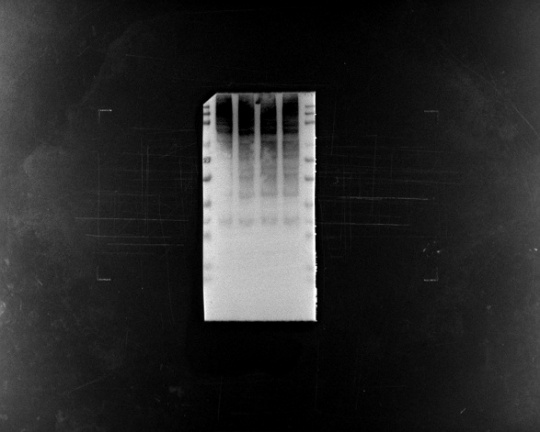

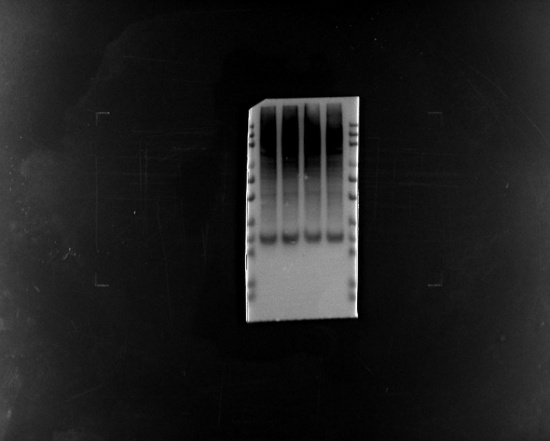
Input-UB:**

**
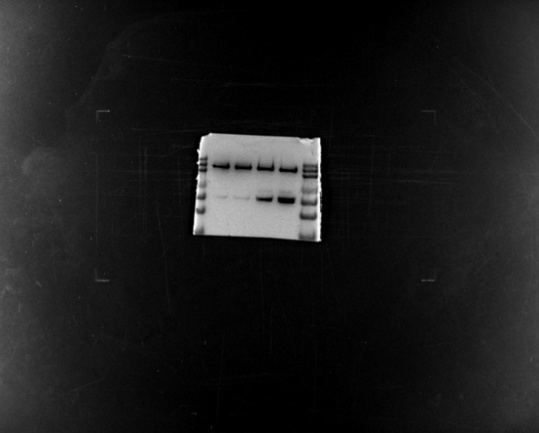

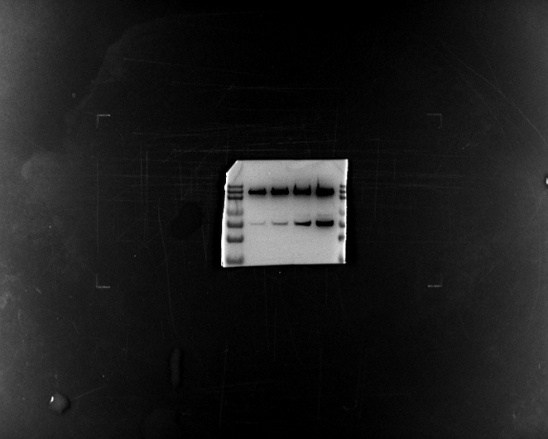
Input-cleaved-TLR3:**

**
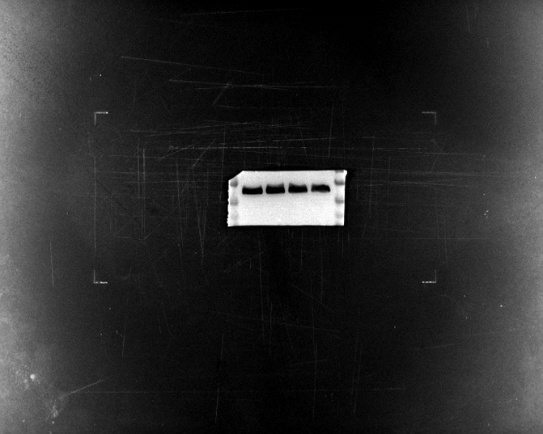

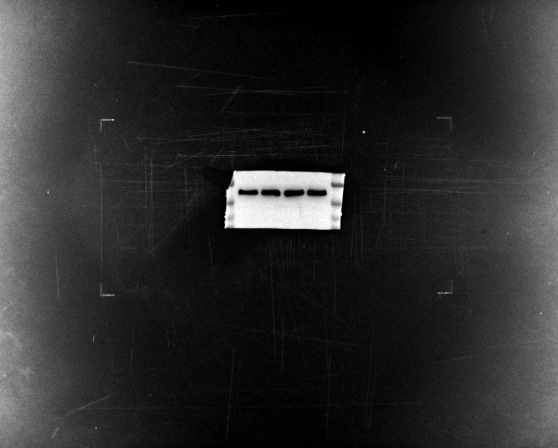
Input-GAPDH:**

**
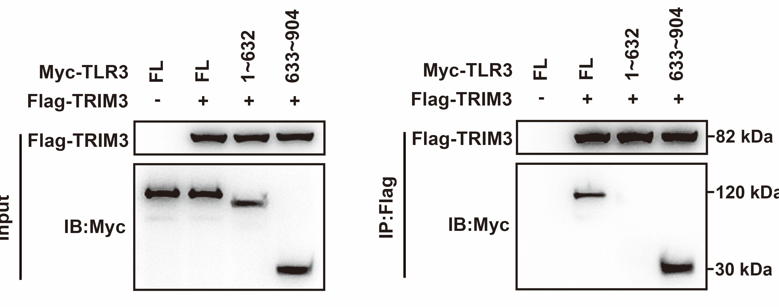
Fig. 5E**

**
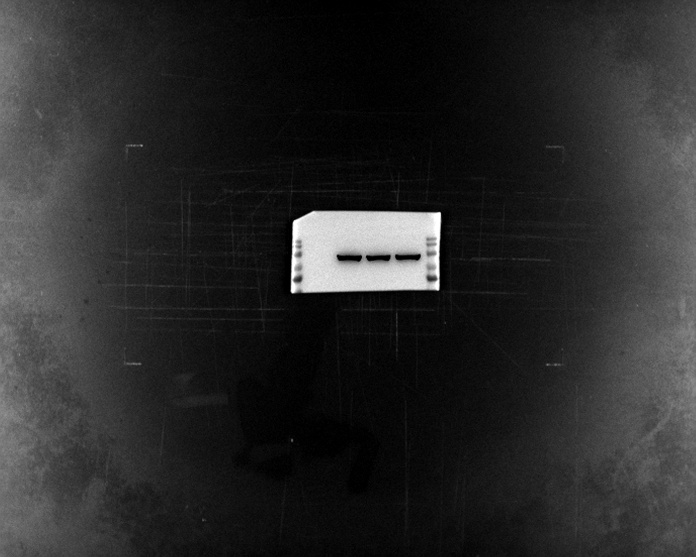
Input-TRIM3:**

**
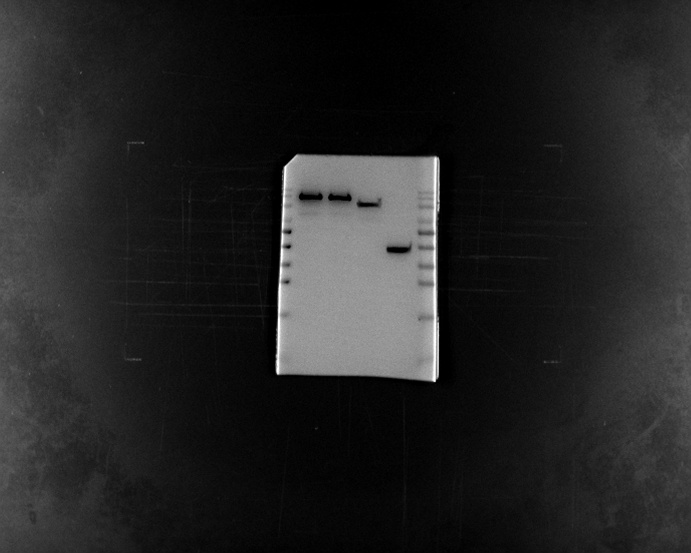
Input-Myc:**

**
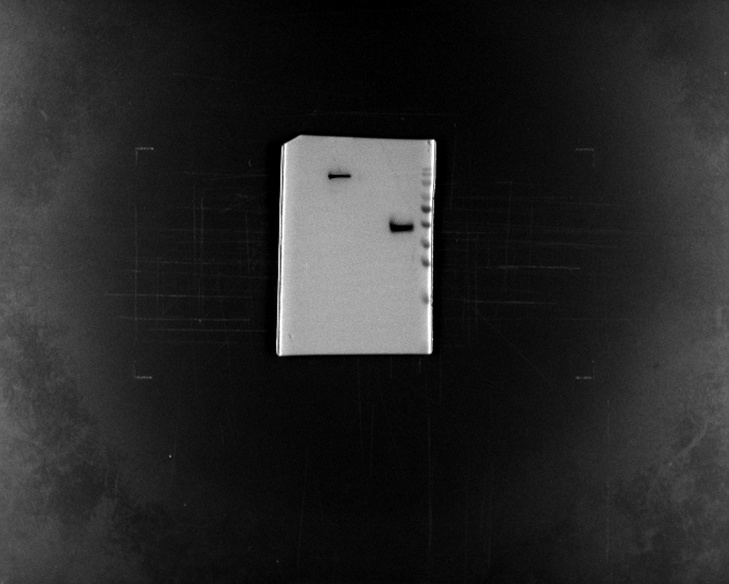

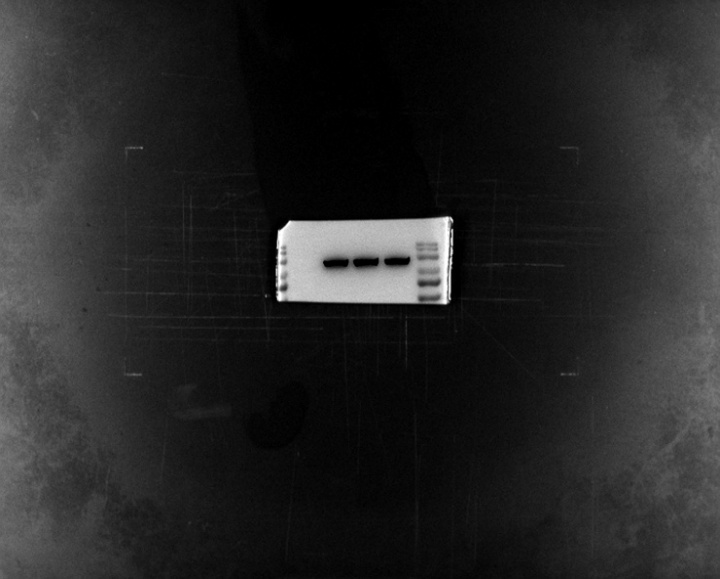
IP-TRIM3:**

**IP-Myc:**

**
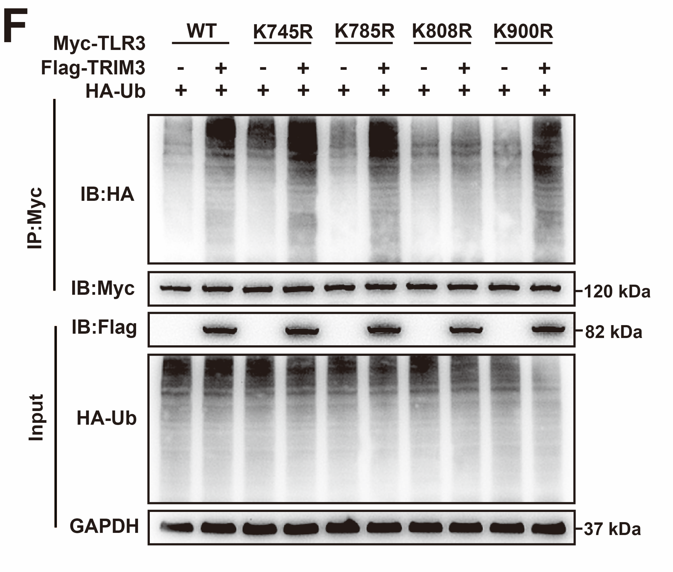
Fig. 5F**

**
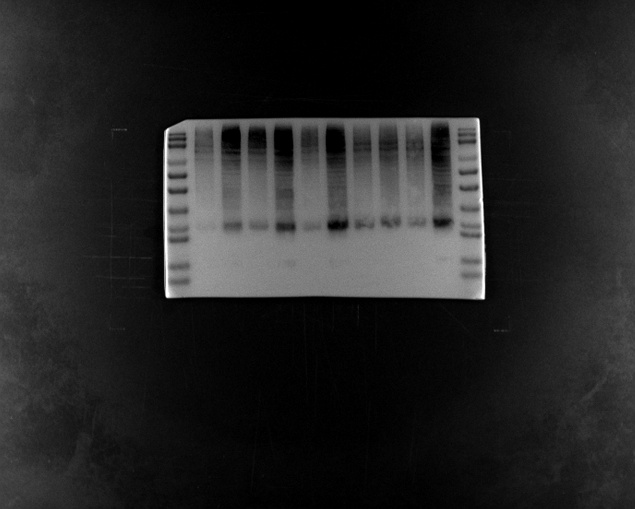
IP-UB:**

**IP-Myc:**

**Input-flag:**

**Input-UB:**

**GAPDH:**

**Fig. S2B:**

**TRIM3:**

**GAPDH:**

**Fig. S2D:**

**TRIM3:**

**GAPDH：**

**Fig. S2E:**

**TRIM3:**

**GAPDH:**

**Fig. S2F:**

**TRIM3:**

**GAPDH:**

**Fig. S5A**

**Fig. S5B**

**Fig. S5C**

**Fig. S5D**

**Fig. S7A**

**TLR3：**

**GAPDH：**

**Fig. S7B**

**TLR3:**

**GAPDH:**

**Fig. S9**

**TRIM3:**

**GAPDH:**
