# Supplementary material for: The TRIM3/TLR3 axis overrides IFN-β feedback inhibition to suppress NSCLC progression
Source: Cell Death Dis. 2026 Jan 16;17(1):44. doi: 10.1038/s41419-025-08265-w (PMC12811290; doi:10.1038/s41419-025-08265-w)
Supplement: Supplementary file 2 — Supplementary Figures [file 41419_2025_8265_MOESM2_ESM.pdf]

SUPPLEMENTARY FIGURES

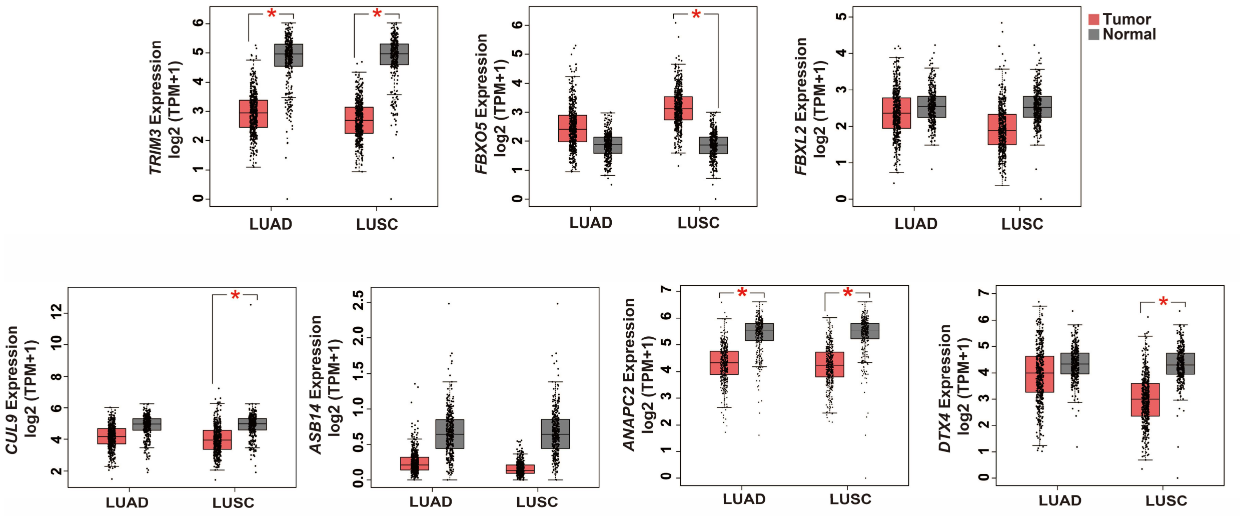

Fig. S1 Differential expression of seven interferon-responsive candidates in lung adenocarcinoma (LUAD) and squamous cell carcinoma (LUSC) compared to normal tissues. *$p < 0.05$.

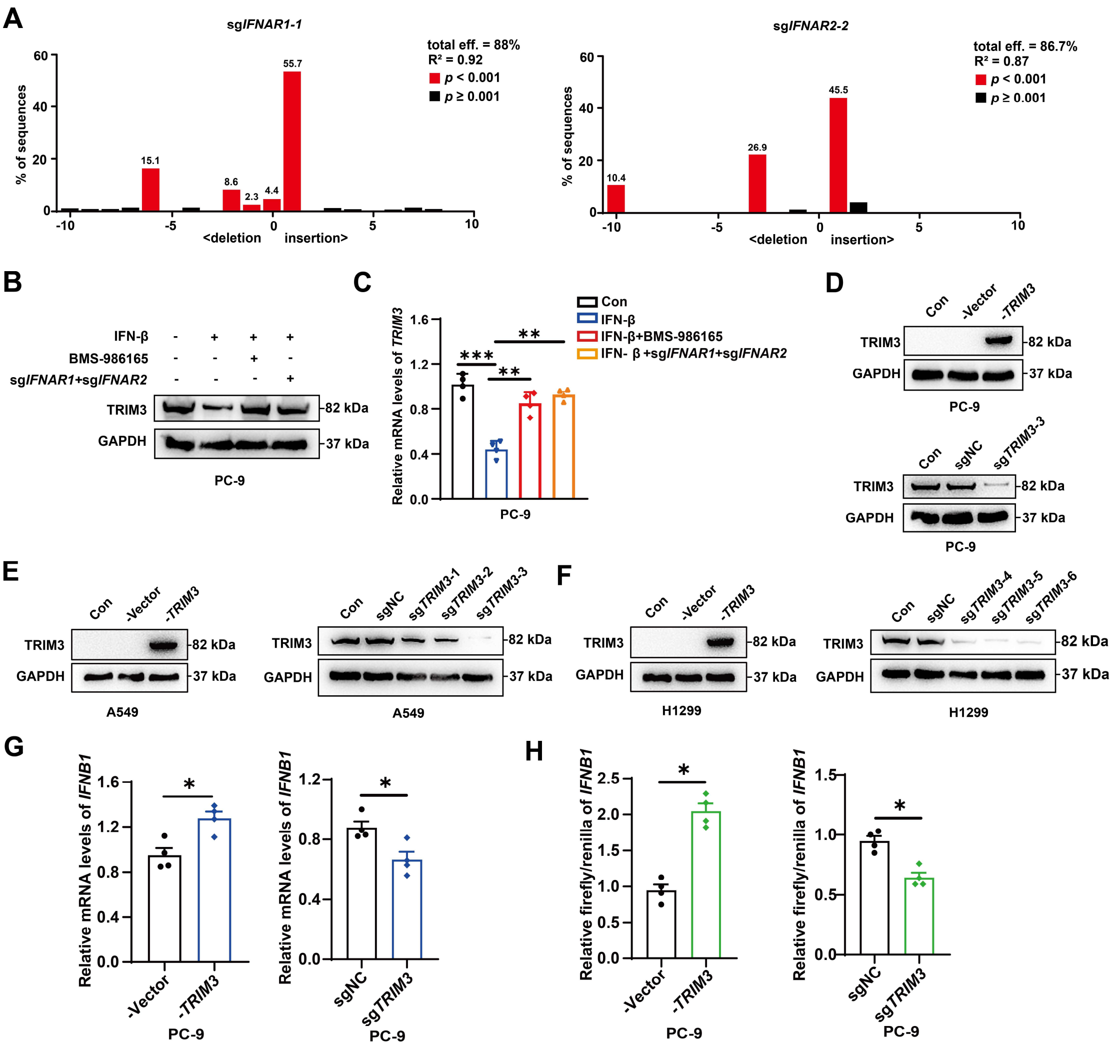

Fig. S2 A Tracking of Indels by DEcomposition (TIDE) analysis of *IFNAR1* and *IFNAR2* knockout efficiency. **B–C** TRIM3 protein and mRNA levels were measured in PC-9 cells treated with IFN-β ± 20 ng/mL BMS-986165 or knockout of *IFNAR1* and *IFNAR2*. Statistical significance was analyzed using one-way ANOVA. **D–F** Western blotting was performed to evaluate the lentiviral knockout and overexpression efficiency of TRIM3 in PC-9, A549 and H1299 cells. **G** *IFNB1* mRNA levels were quantified by qPCR in PC-9 cells with TRIM3 modulation (n=4). Significance was assessed using Student's t test. **H** Luciferase reporter assays were performed in PC-9 cells transfected with *IFNB1* promoter luciferase constructs (n=4). Significance was assessed using Student's t test. *$p < 0.05$; **$p < 0.01$; ***$p < 0.001$.

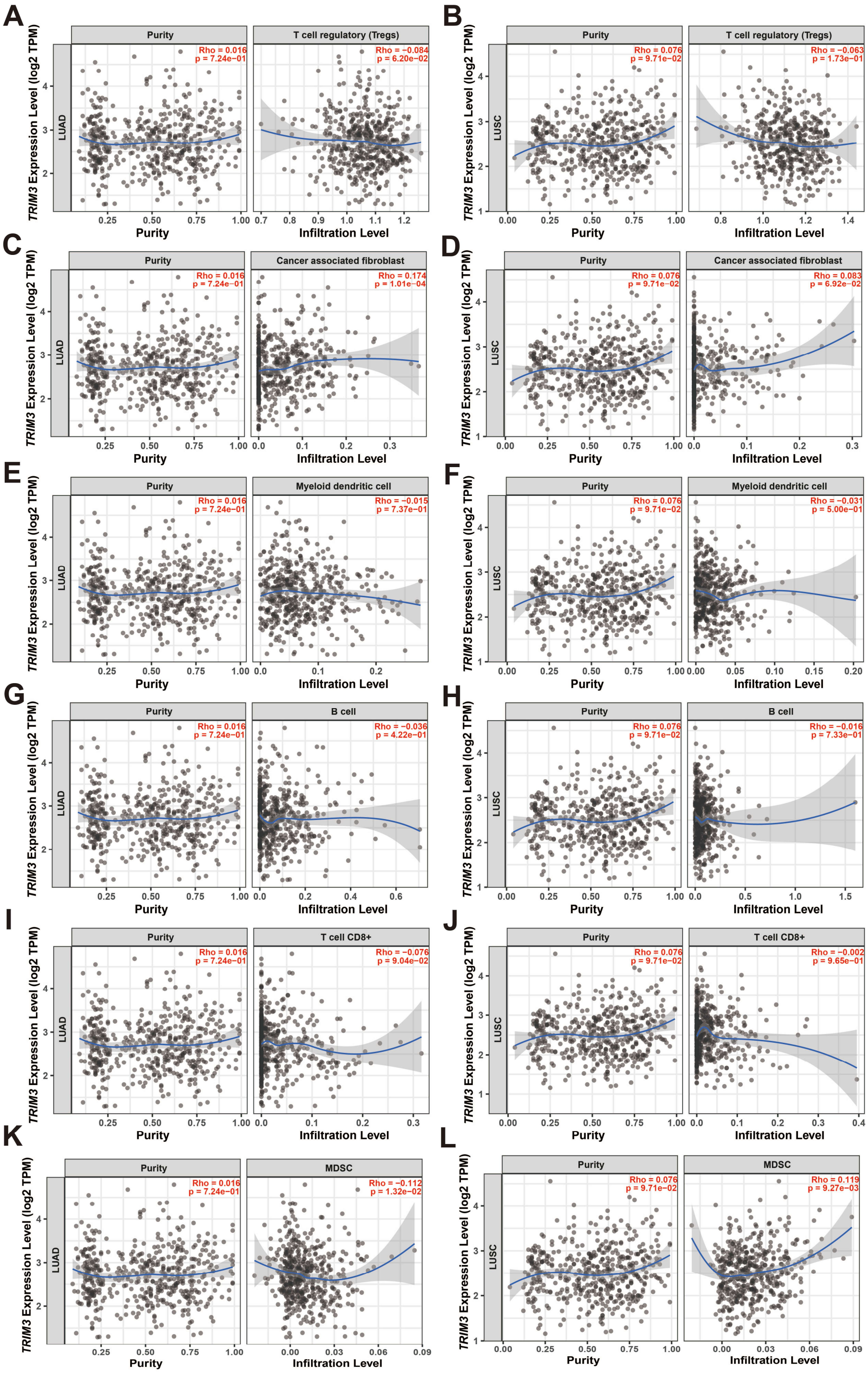

Fig. S3 A–L Bioinformatic analysis of correlations between *TRIM3* expression and infiltration levels of Tregs, cancer-associated fibroblasts, dendritic cells, B cells, CD8^{+} T cells and myeloid-derived suppressor cells in LUAD and LUSC.

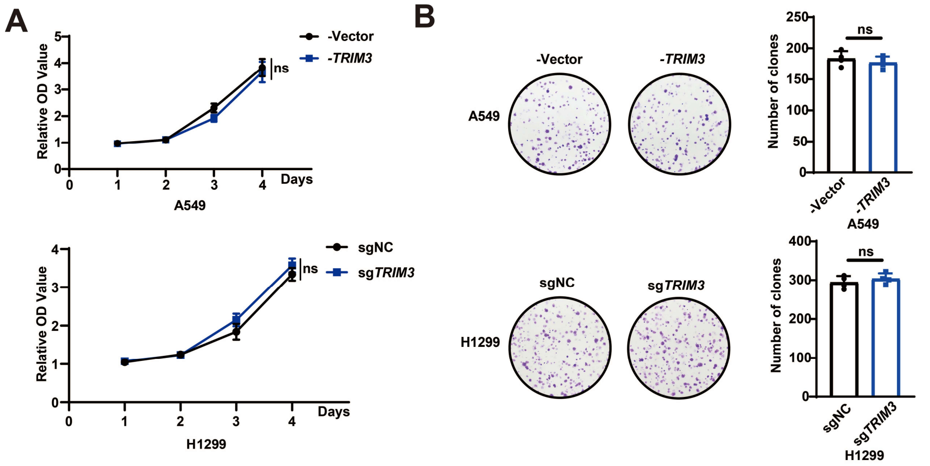

Fig. S4 A–B Colony formation and CCK-8 assays were performed to analyze the impact of TRIM3 modulation on A549 and H1299 cell proliferation. Significance was assessed using Student’s t test. ns = no significance.

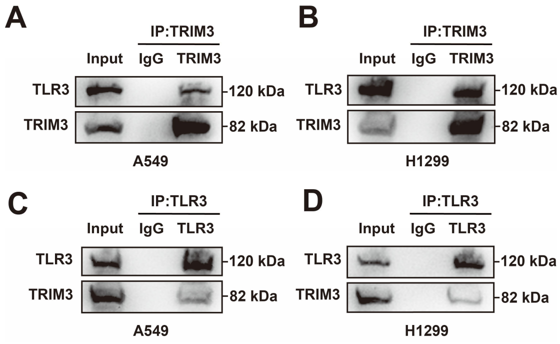

Fig. S5 A–B Co-IP analysis (IP-TRIM3) validated physical interaction between TRIM3 and TLR3 in A549 and H1299 cells. **C–D** Co-IP analysis (IP-TLR3) validated physical interaction between TRIM3 and TLR3 in A549 and H1299 cells.

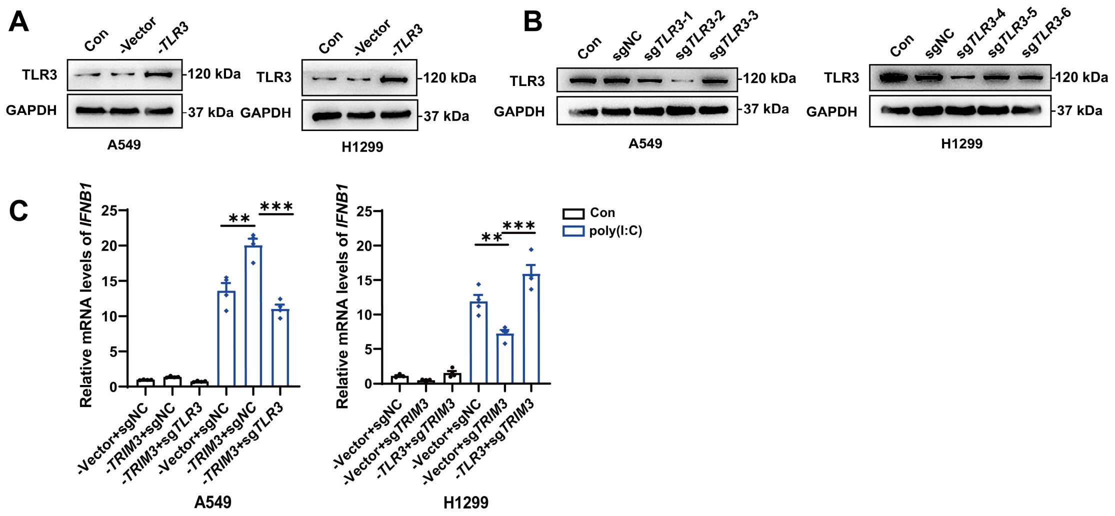

Fig. S6 A–B Western blotting was performed to evaluate the lentiviral overexpression and knockout efficiency of *TLR3* in H1299 and A549 cells. **C** qPCR analysis of *IFNB1* mRNA expression in A549 and H1299 cells (n=4). Statistical significance was analyzed using one-way ANOVA. $^{**}p < 0.01$; $^{***}p < 0.001$.

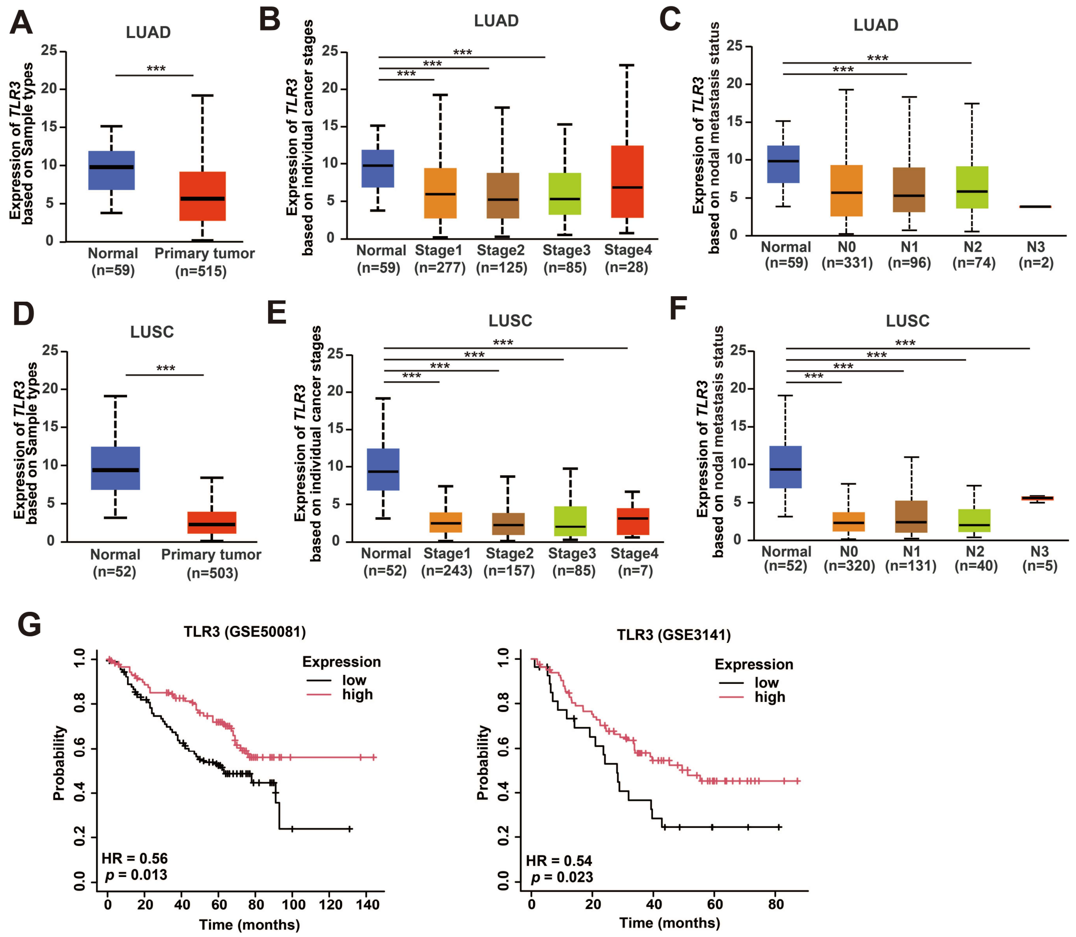

Fig. S7 A–F *TLR3* mRNA levels were analyzed in patients with LUAD and LUSC across tumor-normal paired samples, tumor stages and lymph node metastasis status. **G** Kaplan-Meier survival curves were plotted to correlate *TLR3* mRNA expression with overall survival of patients with NSCLC in GSE50081 and GSE3141. *$p < 0.05$; **$p < 0.01$; ***$p < 0.001$.

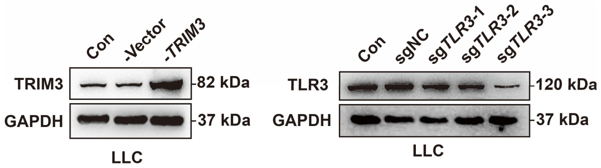

Fig. S8 Western blotting was performed to evaluate the lentiviral overexpression of TRIM3 and knockout efficiency of *TLR3* in LLC cells.

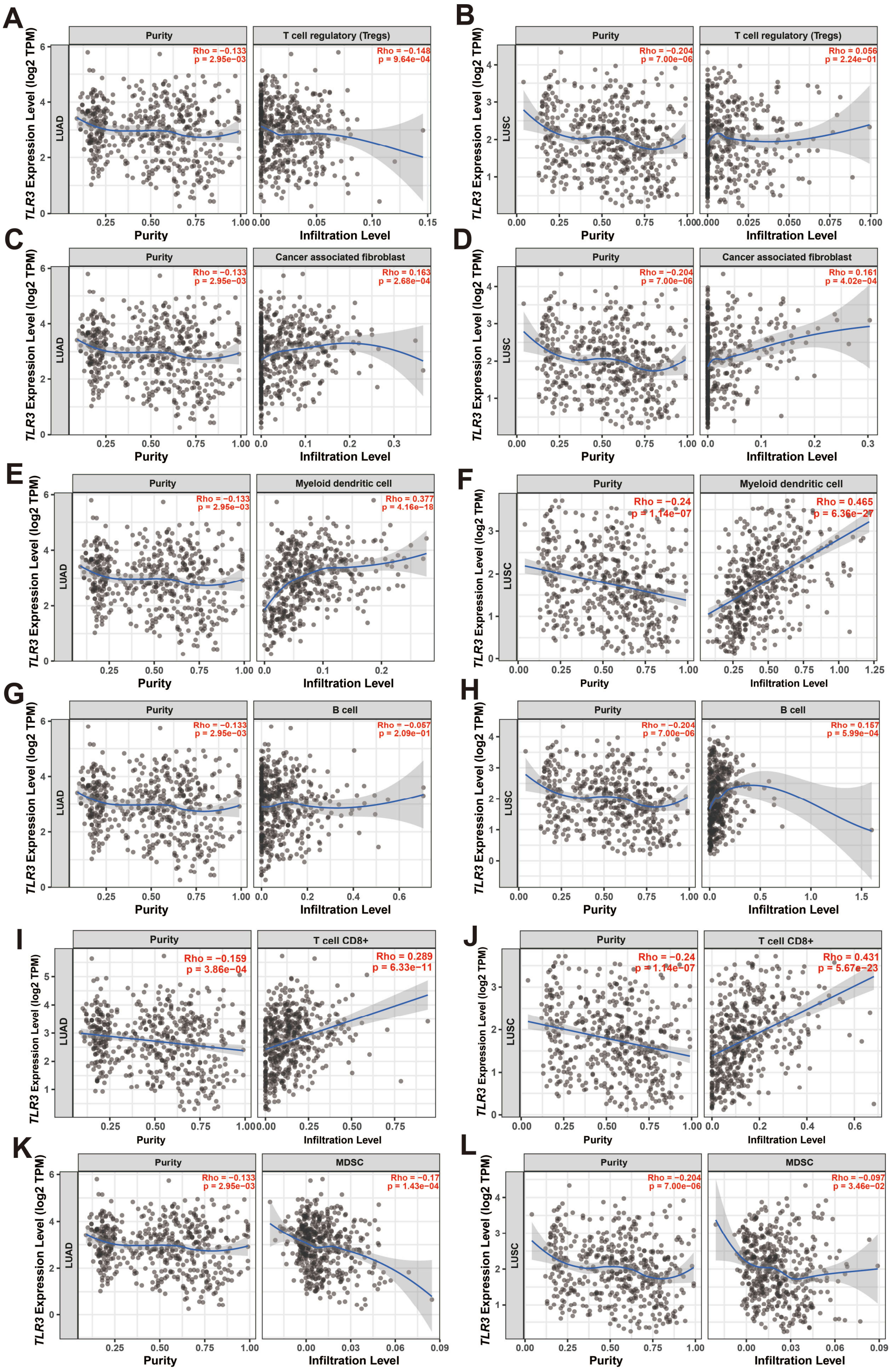

Fig. S9 A–L Bioinformatic analysis of the correlations between *TLR3* expression and infiltration levels of Tregs, cancer-associated fibroblasts, dendritic cells, B cells, $CD8^+$ T cells and myeloid-derived suppressor cells in LUAD and LUSC.
