# Supplementary material for: The TRIM3/TLR3 axis overrides IFN-β feedback inhibition to suppress NSCLC progression
Source: Cell Death Dis. 2026 Jan 16;17(1):44. doi: 10.1038/s41419-025-08265-w (PMC12811290; doi:10.1038/s41419-025-08265-w)
Supplement: Supplementary file 3 — Supplementary Tables [file 41419_2025_8265_MOESM3_ESM.pdf]

Table S1. Primer sequences for sgRNA

Gene symbol	Primer number	Sequences (5' - 3')
Human *TRIM3*	Primer-1-F	accgCGACCTGGAGACCATTTGTG
	Primer-1-R	aaacCACAAATGGTCTCCAGGTCG
	Primer-2-F	accgCCCGACTAAGGCAATTGCTG
	Primer-2-R	aaacCAGCAATTGCCTTAGTCGGG
	Primer-3-F	accgCTCTACGGACAGCCAGTGCG
	Primer-3-R	aaacCGCACTGGCTGTCCGTAGAG
	Primer-4-F	accgGTGTACACAGCGCGCACGGA
	Primer-4-R	aaacTCCGTGCGCGCTGTGTACAC
	Primer-5-F	accgAAGCGCCGTGTCAAGTCCCC
	Primer-5-R	aaacGGGGACTTGACACGGCGCTT
	Primer-6-F	accgTTGCGCACCAGCAACACCTC
	Primer-6-R	aaacGAGGTGTTGCTGGTGCGCAA
Human *TLR3*	Primer-1-F	accgTCAACGACTGATGCTCCGAA
	Primer-1-R	aaacTTCGGAGCATCAGTCGTTGA
	Primer-2-F	accgATAACAACTTAGCACGGCTC
	Primer-2-R	aaacGAGCCGTGCTAAGTTGTTAT
	Primer-3-F	accgCAGCTAACTAGCTTGGATGT
	Primer-3-R	aaacACATCCAAGCTAGTTAGCTG
	Primer-4-F	accgCTCTGGAAACACGCAAACCC
	Primer-4-R	aaacGGGTTTGCGTGTTTCCAGAG
	Primer-5-F	accgGGAAATAAATGGGACCACCA
	Primer-5-R	aaacTGGTGGTCCCATTTATTTCC
	Primer-6-F	accgCCAATTGCGTGAAAACACCC
	Primer-6-R	aaacGGGTGTTTTCACGCAATTGG
Mouse *TLR3*	Primer-1-F	accgATGTTAGAGGGAAGATCATC
	Primer-1-R	aaacGATGATCTTCCCTCTAACAT
	Primer-2-F	accgACATTAGATCGAGTTCTGTC
	Primer-2-R	aaacGACAGAACTCGATCTAATGT
	Primer-3-F	accgGTTGGGCGTTGTTCAAGAGG
	Primer-3-R	aaacCCTCTTGAACAACGCCCAAC

Table S2. Primer sequences for sgRNA

Gene symbol	Primer number	Sequences (5' - 3')
Human *IFNAR1*	Primer-1-F	accgAGTGGATAATCCTGGATCAC
	Primer-1-R	aaacGTGATCCAGGATTATCCACT
	Primer-2-F	accgTGATACTGAAATACAAGGTA
	Primer-2-R	aaacTACCTTGTATTTCAGTATCA
	Primer-3-F	accgAAAGAACTGGGATGGATAAT
	Primer-3-R	aaacATTATCCATCCCAGTTCTTT
Human *IFNAR2*	Primer-1-F	accgAATGACCTCCACCATATCCA
	Primer-1-R	aaacTGGATATGGTGGAGGTCATT
	Primer-2-F	accgATAGTGACACTGAAATGGAT
	Primer-2-R	aaacATCCATTTCAGTGTCACTAT
	Primer-3-F	accgAAACCAACAATCTCAAACTC
	Primer-3-R	aaacGAGTTTGAGATTGTTGGTTT

Table S3. Primer sequences for qPCR

Gene symbol	Sequences (5' - 3')
Human *TRIM3*	GGCAAACGAAAGGACAACCC
	CACTGGTTGTTGCTGTCTGC
Human *IFNB1*	TCTCCTGTTGTGCTTCTCCAC
	GCCTCCCATTCAATTGCCAC
Human *GAPDH*	GACAGTCAGCCGCATCTTCT
	GCGCCCAATACGACCAAATC
